# Combined heterozygosity of *FLT3*^ITD^, *TET2*, and *DNMT3A* results in aggressive leukemia

**DOI:** 10.1172/jci.insight.162016

**Published:** 2022-09-08

**Authors:** Baskar Ramdas, Palam Lakshmi Reddy, Raghuveer Singh Mali, Santhosh Kumar Pasupuleti, Ji Zhang, Mark R. Kelley, Sophie Paczesny, Chi Zhang, Reuben Kapur

**Affiliations:** 1 Department of Pediatrics, Herman B Wells Center for Pediatric Research, Indiana University School of Medicine, Indianapolis, Indiana, USA.; 2 Department of Microbiology and Immunology, Hollings Cancer Center, Medical University of South Carolina, Charleston, South Carolina, USA.; 3 Department of Medical and Molecular Genetics,; 4 Department of Molecular Biology and Biochemistry, and; 5 Department of Microbiology and Immunology, Indiana University School of Medicine, Indianapolis, Indiana, USA.

**Keywords:** Hematology, Cytokines, Epigenetics, Monocytes

## Abstract

Heterozygous mutations in *FLT3^ITD^*, *TET2*, and *DNMT3A* are associated with hematologic malignancies in humans. In patients, cooccurrence of mutations in *FLT3*^ITD^ combined with *TET2* (*TF*) or *FLT3*^ITD^ combined with *DNMT3A* (*DF*) are frequent. However, in some rare complex acute myeloid leukemia (AML), all 3 mutations cooccur — i.e., *FLT3*^ITD^, *TET2*, and *DNMT3A* (*TFD*). Whether the presence of these mutations in combination result in quantitative or qualitative differences in disease manifestation has not been investigated. We generated mice expressing heterozygous *Flt3*^ITD^ and concomitant for either heterozygous loss of *Tet2* (*TF*) or *Dnmt3a* (*DF*) or both (*TFD*). *TF* and *DF* mice did not induce disease early on, in spite of similar changes in gene expression; during the same time frame, an aggressive form of transplantable leukemia was observed in *TFD* mice, which was mostly associated with quantitative but not qualitative differences in gene expression relative to *TF* or *DF* mice. The gene expression signature of *TFD* mice showed remarkable similarity to the human *TFD* gene signature at the single-cell RNA level. Importantly, *TFD*-driven AML responded to a combination of drugs that target *Flt3*^ITD^, inflammation, and methylation in a mouse model, as well as in a PDX model of AML bearing 3 mutations.

## Introduction

Recent whole genome and exome sequencing studies have pointed to substantial genetic heterogeneity among patients with acute myeloid leukemia (AML). These studies have also revealed examples of rare but complex AML with cooccurrence of many mutations. Depending upon the specific combination, various patterns of such combination mutations have been described in the literature (1, 2, 3, 4). Frequently, these AML result in poor clinical outcomes, including bad prognosis and reduced long-term survival. It is possible that combined molecular effects of multiple oncogenic mutations may have unpredictable consequences that can influence cellular phenotype, clinical behavior, and responsiveness to therapy. Although rare and unique in their existence, AML driven by 3 or more mutations should be given special consideration. We know very little about the mechanistic interactions between different mutations in AML carrying 3 or more mutations. It is unclear if AML with 3 mutations induce a set of genes that are unique to them and are qualitatively different from those AML with 2 mutations, or if something else drives their development, including perhaps quantitative differences in the gene expression.

Currently available standard-of-care regimens for AML fail to cure this debilitating disease in patients. Hematopoietic stem cell transplantation (HSCT) is the only curative approach; however, this approach isn’t full proof and is frequently associated with high morbidity and mortality. To date, several efforts have been made to predict therapy-related outcomes and their association with patient cytogenetic background. Patients with normal cytogenetics (CN) make up approximately half of all AML cases. Our understanding of the molecular changes in various patients with AML is critical not only to understand the mechanisms leading to pathological changes, but also for risk stratification and for deciding optimal therapy for these patients. In this regard, molecular aberrations in genetic and epigenetic regulators, such as *DNMT3A*, *TET2*, *NPM1*, and *FLT3^ITD^*, have been investigated in some detail. In a significant number of normal CN AML patients, cooccurrence of 2 of these mutations is found quiet frequently. For instance, *FLT3^ITD^ TET2* (1, 5), *FLT3^ITD^* *DNMT3A* (2), *DNMT3A TET2* (6, 7), or *FLT3^ITD^ NPM1* (8) mutations frequently coexist in normal CN patients with AML (9–11). The cooccurrence of 2 or 3 mutations shows a significant impact on prognosis, including risk of relapse and overall survival in these patients. Presence of *TET2* mutations, along with mutated *NPM1* without *FLT3^ITD^* mutation, results in a lower complete remission rate, shorter disease, and event-free survival (3, 12). In some cases, patients harboring up to 3 of these mutations have been shown to be associated with higher WBC counts (4). In other situations, individuals with either 2 mutations (*DNMT3A FLT3* or *DNMT3A NPM1*) or 3 mutations (*DNMT3A:FLT3:NPM1*) result in similar overall survival (13). Interestingly, cooccurrence of *IDH1/2* mutations with *NPM1*, without *DNMT3A* mutations, do not have an inferior overall survival, suggesting the significance of comutations in determining prognosis. Cooccurrence of *TET2 FLT3^ITD^ DNMT3A* mutations in a subset of AML is associated with poor outcomes, relapse, and resistance to standard therapies. However, it is unclear if cooccurrence of these 3 mutations has a redundant effect, an additive effect, or a synergistic effect or if they induce novel pathways compared with cooccurrence of only 2 mutations. Furthermore, it is also unclear how the kinetics of disease differ qualitatively and quantitatively between AML with cooccurrence of 2 and/or 3 of these above described mutations. In the current study, we have analyzed and compared the genetic, phenotypic, and transcriptomic features of AML driven by a combination of *FLT3^ITD^ TET2*, *FLT3^ITD^* *DNMT3A*, or *FLT3^ITD^ TET2 DNMT3A*, utilizing AML models, patient cells, and transcriptomic data derived from both mice and humans.

## Results

### Combined heterozygous loss of Tet2 (Tet2^+/–^) and Dnmt3a (Dnmt3a^+/–^) and expression of Flt3^ITD/WT^ (Tet2^+/–^ Dnmt3a^+/–^ Flt3^ITD/WT^) results in an aggressive lethal myeloid leukemia.

**Figure 1:**
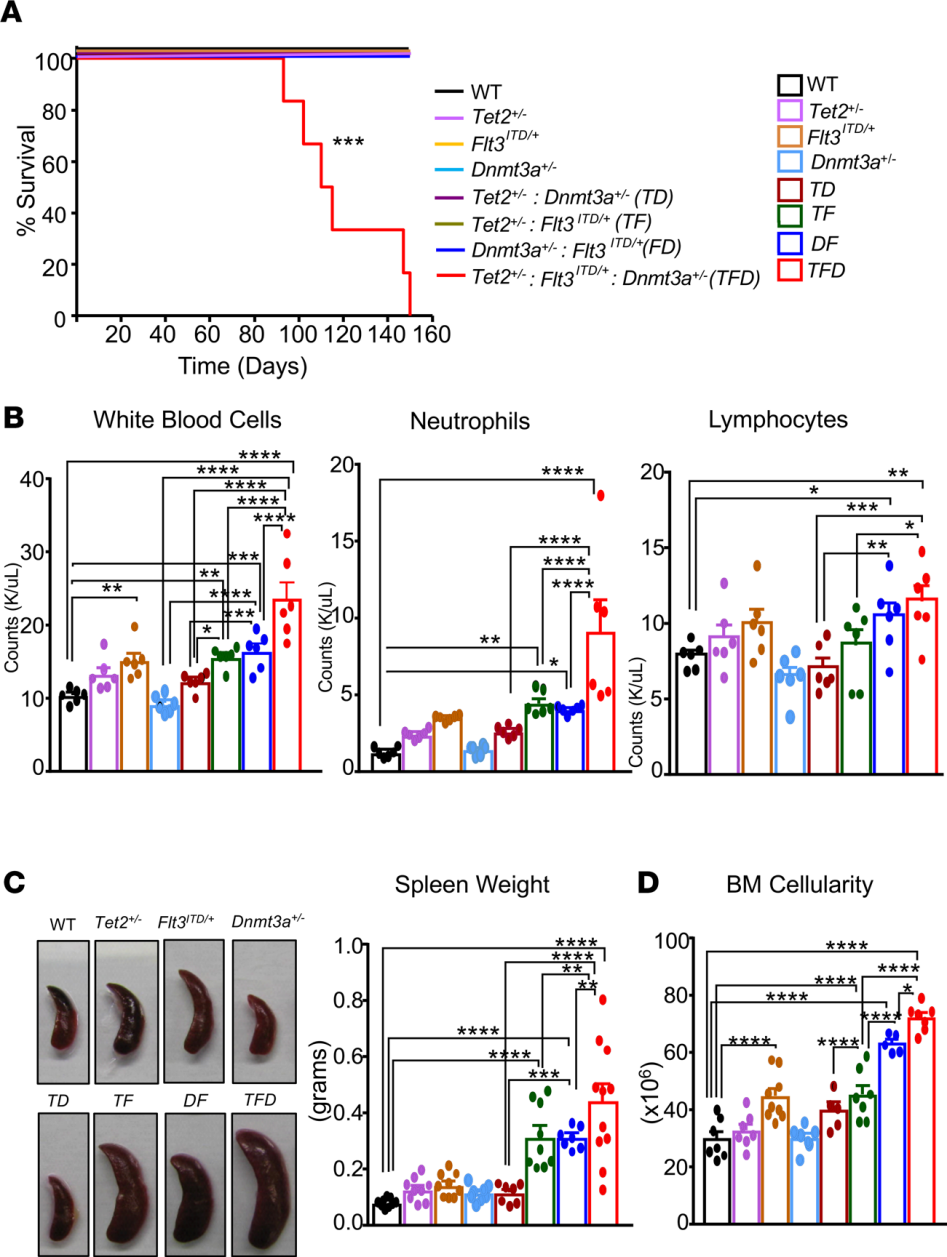
Combined heterozygous loss of *Tet2* and *Dnmt3a* and expression of *Flt3*^ITD/WT^ causes aggressive lethal myeloid leukemia in mice. (**A**) WT, *Tet2^+/–^*, *Flt3*^ITD/WT^, *Dnmt3a^+/–^*, *Tet2^+/–^* *Flt3*^ITD/WT^, *Dnmt3a^+/–^* *Flt3*^ITD/WT^, *Tet2^+/–^ Dnmt3a^+/–^*, and *Tet2^+/–^ Dnmt3a^+/–^ Flt3*^ITD/WT^ mice were treated with poly (I:C) and monitored for acute myeloid leukemia progression and survival. Shown is Kaplan Meir survival curve (*n =* 5–6 mice in each group). (**B**) PB counts (K/μL): WBCs, neutrophils, and lymphocytes after 2 months of poly(I:C) treatment. (**C**) Representative spleen pictures from indicated genotypes and consolidated spleen weights from 2 independent experiments. (**D**) Consolidated BM cellularity from 2 independent experiments. Each data point represents a value from an individual mouse. Data are shown as mean ± SEM. Statistical analysis was performed using GraphPad version 7 using 1-way ANOVA with uncorrected Fisher’s test. *P < 0.05, **P < 0.01, ***P < 0.001, ****P < 0.0001.

Given that the cooccurrence of mutations in *TET2*, *DNMT3A*, and *FLT3^ITD^* in patients is associated with aggressive AML and with relapse, we assessed the biological consequences of coexistence of these mutations on various hematologic parameters. We hypothesized that the presence of these mutations, either in combination of 2 or 3, is likely to result in both qualitative and quantitative different outcomes with regard to the development of AML. Analysis of OHSU and TCGA (https://www.cbioportal.org/) data demonstrates the frequent presence of concurrent mutations in *DNMT3A*, *FLT3*, and *TET2* genes in patients with AML. To model how the combination of these human AML mutations manifests when present in various combinations, we bred mice to obtain 8 distinct genotypes: WT, single heterozygous for *Tet2^+/–^*, *Flt3^ITD/WT^*, and *Dnmt3a^+/–^*; double heterozygous for *Tet2^+/–^ Dnmt3a^+/–^* (*TD*), *Tet2^+/–^ Flt3^ITD/WT^*(*TF*), and *Dnmt3a^+/–^ Flt3^ITD/WT^* (*DF*); and triple heterozygous for *Tet2^+/–^ Flt3^ITD/WT^ Dnmt3a^+/–^* (*TFD*). Given that *Dnmt3a* is a floxed conditional allele, all mice at 8–10 weeks of age were treated with poly(I:C) and monitored for survival. Of all 8 genotypes examined, only *TFD* mice succumbed by 150 days, as shown in Figure 1A. We next assessed all 8 genotypes for hematologic parameters 2 months after poly(I:C) treatment. As seen in Figure 1B, peripheral blood (PB) WBC counts were significantly higher in triple-mutant *TFD* mice compared with any of the other 7 genotypes. A modest but a significant increase in PB cell count was also seen in double mutants *TF* and *DF* compared with WT controls. Likewise, PB neutrophil counts were also highest in *TFD* mice compared with other genotypes (Figure 1B). A modest increase in lymphocyte count was observed in *TFD* and *DF* mice relative to other 6 genotypes (Figure 1B). Representative spleen pictures and the quantitation of spleen weights are shown in Figure 1C, indicating spleen size and weight were significantly and equally elevated in double mutant *TF* and *DF* mice compared with WT, while triple-mutant *TFD* mice showed significantly higher spleen weight relative to all other 7 genotypes. Next, we assessed BM cellularity in the 8 genotypes. As shown in Figure 1D, significantly higher BM cellularity was seen in double-mutant *TF* and *DF* mice compared with WT control, although the *TFD* mice showed the highest cellularity relative to all other genotypes. In summary, these results demonstrate that the presence of *TFD* mutations quantitatively changes various hematopoietic parameters in mice, resulting in a more aggressive phenotype within a similar time frame and age compared with *TF* and *DF* mice from the same group.

**Figure 2:**
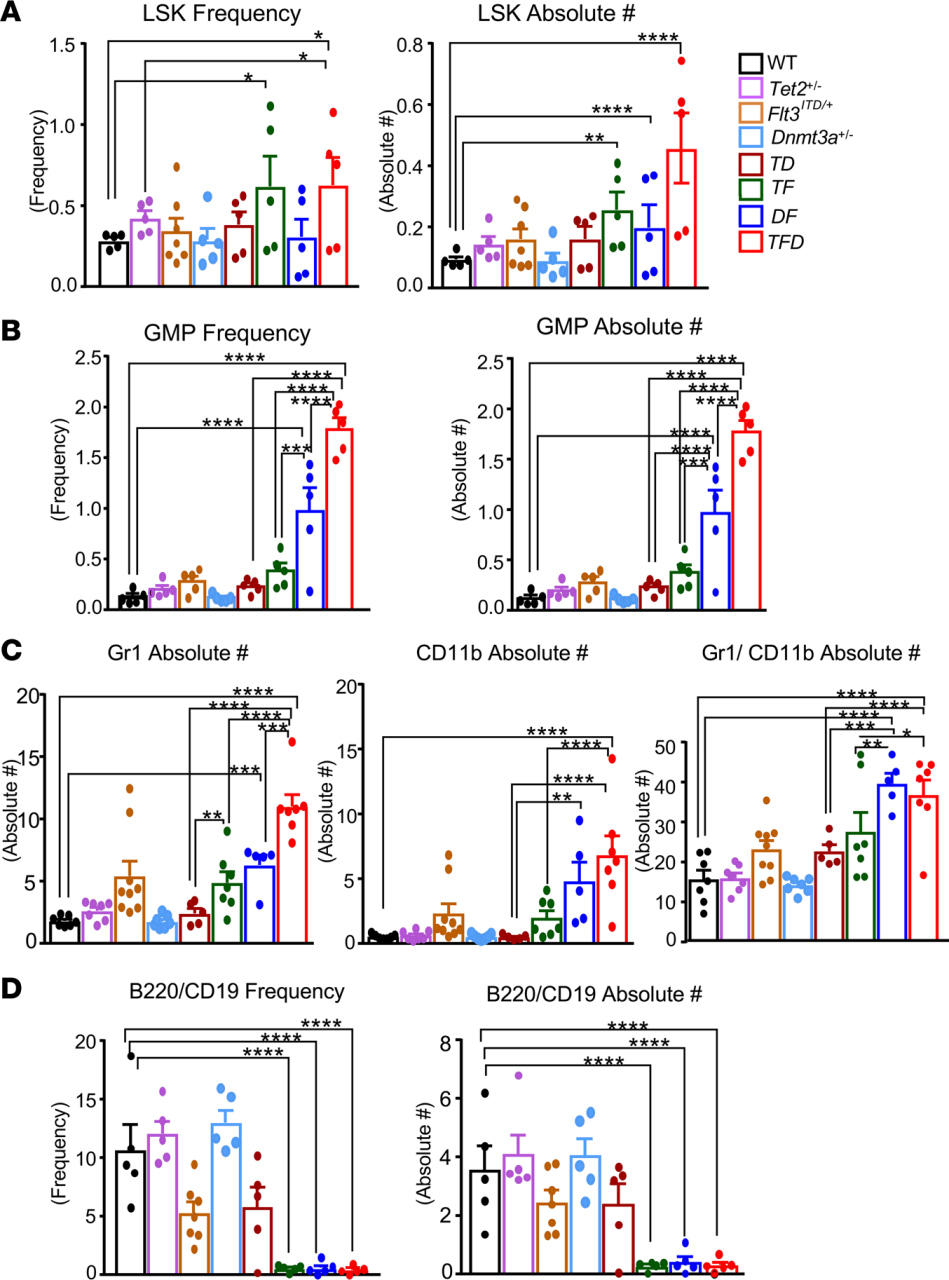
Analysis of immature and mature cells in the BM of *Tet2^+/–^ Dnmt3a^+/–^ Flt3*^ITD/WT^ mice. (**A**) WT, *Tet2^+/–^*, *Flt3*^ITD/WT^, *Dnmt3a^+/–^*, *Tet2^+/–^ Dnmt3a^+/–^*, *Tet2^+/–^* *Flt3*^ITD/WT^, *Dnmt3a^+/–^* *Flt3*^ITD/WT^, and *Tet2^+/–^ Dnmt3a^+/–^ Flt3*^ITD/WT^ mice were treated with poly (I:C) as described in Figure 1. Immunophenotyping of immature and mature cells was performed using various cell surface markers followed by flow cytometry. (**A**) Frequency and absolute number of Lin-Sca1^+^KIT^+^ cells in the BM. Consolidated data are from 2 independent experiments. (**B**) Frequency and absolute number of GMP cells in the BM. Consolidated data are from 2 independent experiments. (**C**) Absolute number of Gr-1^+^ cells, CD11b^+^ cells, and Gr-1/CD11b–double-positive myeloid cells in the BM of indicated genotypes. (**D**) Frequency and absolute number of B220/CD19–double-positive lymphoid cells in the BM of indicated genotype. Consolidated data are from 3 independent experiments. Each data point represents a value from an individual mouse. Data are shown as mean ± SEM. Statistical analysis was performed using GraphPad version 7 using 1-way ANOVA with uncorrected Fisher’s test. *P < 0.05, **P < 0.01, ***P < 0.001, ****P < 0.0001.

We next assessed how these mutations cooperate within the more immature progenitor cell compartment of the BM. We first assessed the frequency of leukemia stem cell (LSC) containing LSK population and found this to be elevated in *TF* and *TFD* mice relative to WT controls (Figure 2A). Likewise, the absolute number of LSK cells were highest in the *TFD* mice relative to any other group, although *TF* and *DF* mice also showed a modest increase relative to WT controls (Figure 2A). A downward trend in the frequency of MEPs was observed in the *TFD* mice relative to any other genotype (data not shown). A significant and comparative increase in the absolute number of common myeloid progenitors (CMPs) was also observed in *TF*, *DF*, and *TFD* mice relative to any other genotype (data not shown). Importantly, an increase in the frequency and absolute number of granulocyte-macrophage progenitors (GMPs) (Figure 2B) was consistently observed in *TF*, *DF*, and *TFD* mice relative to WT and single-mutant mice, although the frequencies and absolute numbers were greatest in *TFD* mice. Taken together, while no significant qualitative differences in the frequencies and absolute numbers of BM progenitors were observed between the double (*TF* and *DF*) and the triple (*TFD*) mutant mice, the absolute number of LSKs and GMPs were quantitatively greater in *TFD* mice relative to *TF* or *DF* mutants. The absolute number of GMPs in *TFD* BM were 4.7-fold and 1.8-fold greater in number compared with *TF* and *DF* mice, respectively (Figure 2B).

Next, we assessed the impact of these mutations on the development of more mature myeloid progenitors in the BM. An increase in absolute number of Gr-1^+^ cells was observed in the BM of *TF*, *DF*, and *TFD* mice relative to WT and single-mutant mice (Figure 2C). A significant increase in the absolute number of CD11b^+^ population was also seen in the BM of *DF* and *TFD* mice relative to other groups (Figure 2C). An increase in the absolute number of Gr-1/CD11b–double-positive cells in the BM was observed in *DF* and *TF* mice, as well as *TFD* mice, relative to other groups. The increase in the frequency of CD11b^+^ and Gr-1/CD11b–double-positive myeloid cells, however, was the highest in the spleen of *TFD* mice (Supplemental Figure 1A; supplemental material available online with this article; https://doi.org/10.1172/jci.insight.162016DS1). We also examined the frequency and absolute number of B cells as assessed by the presence of B220/CD19–double-positive cells in the BM. As seen in Figure 2D, while the composition of myeloid cells in the BM of *TFD* mice was quantitatively distinct from *TF* and *DF* mice, the impact on B cell lineage was similar between *TF*, *DF*, and *TFD* mice (Figure 2D). In all 3 genotypes, a similar and significant reduction in the frequency and absolute number of B220/CD19–double-positive lymphoid cells was noted in the BM, as well as in the spleen (Figure 2D and Supplemental Figure 1B). Thus, in lymphoid cells, loss of 2 alleles of epigenetic regulators, *Tet2* or *Dnmt3a*, in combination with the expression of *Flt3^ITD/WT^*, results in similar outcomes as loss of 1 allele of an epigenetic regulator and expression of *Flt3^ITD/WT^*. In contrast, in myeloid cells, loss of 2 alleles of epigenetic regulators (*Tet2* and *Dnmt3a*) in combination with the expression of *Flt3^ITD/WT^* has a more profound impact.

### Combined heterozygous loss of Tet2^+/–^ and Dnmt3a^+/–^ and expression of Flt3^ITD/WT^ results in a transplantable myeloid leukemia.

**Figure 3:**
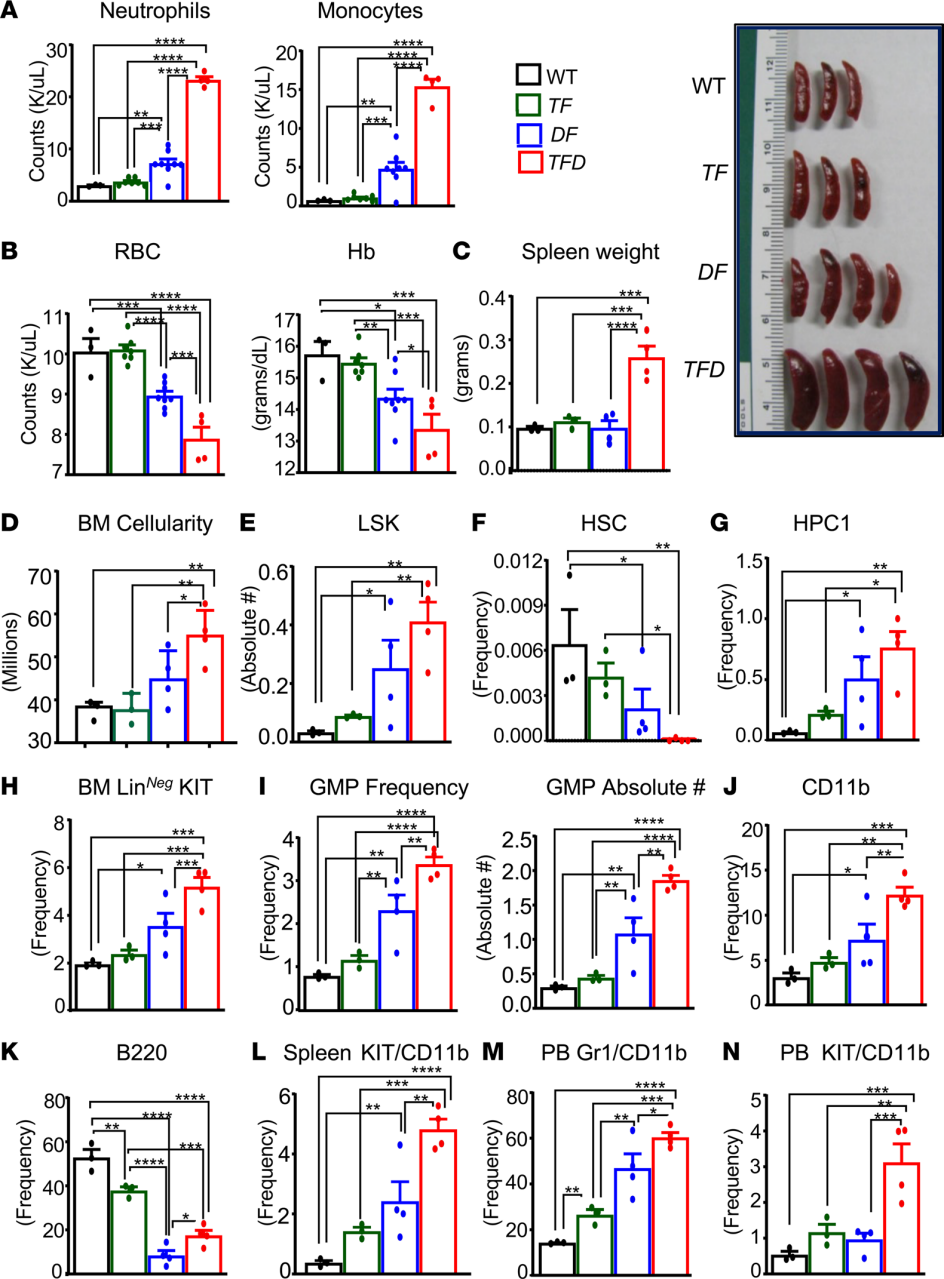
Combined heterozygous loss of *Tet2* and *Dnmt3a* and expression of *Flt3*^ITD/WT^ causes transplantable myeloid leukemia in mice. BM cells (2 × 10^6^ cells) from WT, *Tet2^+/–^* *Flt3*^ITD/WT^, *Dnmt3a^+/–^* *Flt3*^ITD/WT^, and *Tet2^+/–^ Dnmt3a^+/–^* *Flt3*^ITD/WT^ mice were transplanted into lethally irradiated C57BL/6 mice through tail vein and monitored for disease progression. (**A**–**D**) Mice were analyzed when moribund around 7 weeks after transplants for PB neutrophils and monocytes counts (**A**), PB RBC and; Hb (**B**), quantification data on spleen weights and representative spleen pictures from indicated genotypes (**C**), and BM cellularity of indicated genotype recipients (**D**). (**E**) Absolute number of LSK cells in BM. (**F**) Frequency of HSC (CD48^–^CD150^+^ LSK). (**G**) Frequency of HPC1 (CD48^+^CD150^–^ LSK) in the BM of indicated genotype recipients. (**H**) Frequency of lin*^–^*KIT*^+^* myeloid progenitors from BM of the indicated genotype recipients. (**I**) Frequency and absolute number of GMP population in the BM of indicated genotype recipients. (**J**) Frequency of CD11b^+^ myeloid cells in BM. (**K**) Frequency of B220^+^ lymphoid cells in the BM of indicated genotypes. (**L**) Frequency of KIT/CD11b–double-positive cells in spleen. (**M**) Frequency of Gr-1/CD11b–double-positive myeloid cells in PB. (**N**) Frequency of KIT/CD11b–double-positive myeloid blast cells in PB. Each data point represents a value from an individual mouse. Data are shown as mean ± SEM. Statistical analysis was performed using GraphPad version 7 using 1-way ANOVA with uncorrected Fisher’s test. *P < 0.05, **P < 0.01, ***P < 0.001, ****P < 0.0001.

Studies described in Figures 1 and 2 assessed the cooperative role of the presence of 2 or 3 heterozygous genetic and epigenetic mutations on the development of AML and their impact on various hematopoietic lineages in the parental transgenic mice. To more specifically assess if the observed leukemia-associated phenotype seen in *TF*, *DF*, and *TFD* mice is transplantable — and, if transplantable, how rapidly the disease manifests — we transplanted BM cells from WT, *TF*, *DF*, and *TFD* mice into lethally irradiated C57BL/6 host. Transplanted mice were monitored for PB counts, progenitor content, and signs of AML development. At the time of moribund, all mice were analyzed for PB, BM, and spleen cellular composition. As seen in Figure 3A, a significant increase in PB neutrophil counts and monocyte counts were observed in mice transplanted with *TFD* BM compared with any other group. This increase was associated with a significant decrease in RBC counts and Hb levels (Figure 3B). Splenomegaly was also observed in *TFD* recipients relative to other genotypes (Figure 3C). BM cellularity was increased in *DF* and *TFD* recipient mice (Figure 3D). Absolute number of LSK cells were elevated most significantly in *TFD* recipient mice (Figure 3E). In contrast, a complete loss of HSCs, as assessed by the loss of CD150^+^CD48^–^ LSK cells was noticed in *TFD* recipient mice (Figure 3F and Supplemental Figure 2A), which was consistent both at the level of frequency and absolute numbers. A tendency toward reduction in these cell types was also noted in *TF* and *DF* mice (Figure 3F and Supplemental Figure 2A). In contrast, the frequency of HPC1, as assessed by the presence of CD150^–^CD48^+^ LSK cells, was significantly increased in *TFD* mice relative to the other 2 groups, although the increases were also seen in the *DF* and *TF* mice compared with controls (Figure 3G). An increase in the Lin^–^Kit^+^ myeloid progenitors was also observed in the *TFD* mice and in *DF* mice relative to controls (Figure 3H). In contrast, both the frequency and the absolute number of GMPs were elevated in all 3 groups but were elevated most dramatically in *TFD* mice (Figure 3I and Supplemental Figure 2B). Consistent with an increase in myeloid progenitors, the frequency of mature CD11b^+^ myeloid cells in the BM was also enhanced in all 3 groups relative to controls (Figure 3J and Supplemental Figure 2C). Consistently, the B220 population was dramatically reduced in the BM of *TFD* and *DF* mice relative to *TF* mice (Figure 3K). Likewise, the frequency of KIT/CD11b–double-positive blasts in the spleen was the highest in the *TFD* mice relative to other groups (Figure 3L). We also looked to see if the changes seen in the BM and spleen of *TFD* mice were reflected in the PB of these mice. As seen in Figure 3, M and N, both the Gr-1/CD11b–double-positive cells and — in particular — the KIT/CD11b–double-positive myeloid blasts in *TFD* mice were significantly greater compared with *DF* and *TF* mice. Taken together, these results suggest that the myeloid disease is transplantable. The hematopoietic impact of a combination of the presence of 2 or 3 mutations in hematopoietic stem and progenitor cells (HSP/Cs) is qualitatively similar; however, the impact is quantitatively significantly more in hematopoietic stem cells and progenitors (HSC/Ps) bearing a combination of 3 mutations seen in *TFD* mice, followed by *DF* and then the *TF* mice. Remarkably, both the analysis of primary mice (Figures 1 and 2) and the transplanted mice (Figure 3) yielded similar phenotypic outcomes.

### RNA-Seq analysis in BM cells derived from TF, DF, and TFD mutants.

Given that, in both the original transgenic mice and after BM transplantation, the disease manifestation was qualitatively similar between *DF*, *TF*, and *TFD* mutant bearing mice but quantitatively accelerated in the setting of triple heterozygosity (i.e., *TFD*), we wanted to examine if these differences were due to quantitative or qualitative differences in gene expression. To assess this, we performed RNA-Seq analysis on BM cells derived from all 4 genotypes (i.e., WT, *DT*, *TF*, and *TFD*). RNA was extracted from BM that was also used for functional and phenotypic analysis shown in Figures 1 and 2. A multidimensional scaling (MDS) plot of distances between whole transcriptomics profile showed distinct differences between WT versus *DF*, *TF*, and *TFD* mutants (Supplemental Figure 3A). We observed 2,328; 2,168; and 1,787 upregulated genes and 1,861; 1,770; and 1,430 downregulated genes in *DF*, *TF*, and *TFD* versus WT, respectively. The variations in gene expression between *DF*, *TF*, and *TFD* was smaller compared with the differences observed between WT versus *DF*, *TF*, and *TFD*, in which the *TF* and *TFD* groups were more similar to each other compared with the *DF* group (Supplemental Figure 3A). 521, 335, and 290 overexpressed genes and 860, 393, and 101 underexpressed genes were identified when compared between *DF* versus *TF*, *DF* versus *TFD*, and *TF* versus *TFD*, respectively.

### Dysregulated expression of genes involved in HSC self-renewal and cytokines in TF, DF, and TFD HSC/Ps.

**Figure 4:**
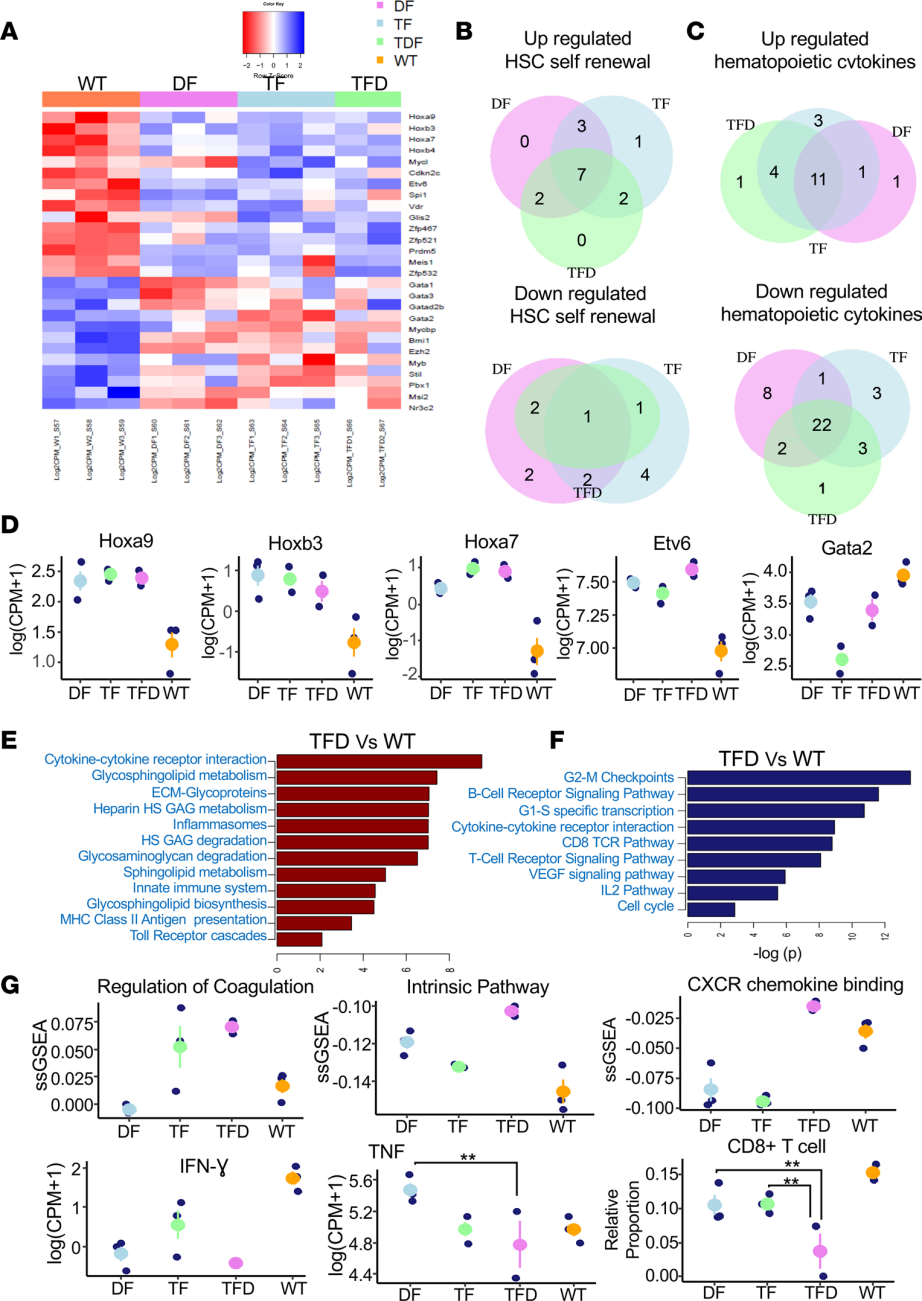
Dysregulated gene expression of HSC self-renewal and hematopoietic cytokines in *TF*, *DF*, and *TFD* leukemia. (**A**) Heatmap showing gene expression profile of dysregulated HSC self-renewal genes in mutants versus WT. (**B**) Venn diagram showing the number of overlap of differentially expressed HSC self-renewal genes between *TF*, *DF*, and *TFD* groups. (**C**) Venn diagram showing the number of overlap of dysregulated hematopoietic cytokines between *TF*, *DF*, and *TFD* mutant groups. (**D**) Plots showing the gene expression of selected HSC self-renewal related genes. In each plot, WT, *DF*, *TF*, and *TFD* are labeled by orange, violet, blue, and green, respectively. (**E**) Pathway enrichment analysis of upregulated genes. (**F**) Pathway enrichment analysis of downregulated genes. (**G**) Differentially expressed genes, cell proportions, and pathways in *TFD* versus *DF* and *TF*. The *y* axes are ssGSEA enrichment score for pathways, log_(CPM+1)_ for gene expression, and relative proportion for cell type.

Pathway enrichment analysis revealed that the genes involved in HSC self-renewal were differentially expressed in all 3 mutant groups versus WT (*P <* 0.05). Fifteen HSC self-renewal genes were upregulated in all 3 mutant groups, out of which 7 were common to all 3 mutant groups — including *Hoxb3*, *Cdkn2c, Myc1*, *Zfp521*, and *Meis1* (14, 15) — and *Zfp532* was upregulated in *DF* and *TF*, *TF* and *TFD*, and *DF* and *TFD* HSC/Ps, respectively, while *Glis2* (16–18) was uniquely upregulated in *TF* group (Figure 4, A–C). In contrast, 12 downregulated genes — *Gata1*, *Gata2*, *Gata3*, *Gatad2b*, *Mycbp*, *Bmi1*, *Ezh2*, *Myb*, *Stil*, *Pbx1*, *Msi2*, and *Nr3c2 —* were identified in all 3 mutant groups (Figure 4, A–C). Figure 4D shows log count per million (CPM) value plots for selective HSC genes related to self-renewal and lineage commitment, including *Hoxa9* (19–21), *Hoxb3*, *Hoxa7*, *Etv6* (22–24), and *Gata2* (25). Other than *Gata2*, most of the genes were similarly modulated in mutant HSC/Ps (Figure 4D). In addition to self-renewal genes, we also observed a significant dysregulation in the expression of hematopoietic cytokines in all 3 mutant groups (Figure 4C). We observed 11 genes — namely, *Csf2ra*, *Cd34*, *Il3ra*, *Csf1r*, *Il6ra*, *Ifngr1*, *Il31ra*, *Ifnar2* (26–29), *Il6st*, *Flt3*, and *Il6* — to be consistently upregulated in all 3 mutant groups (Figure 4B, Supplemental Figure 3B, and Supplemental Table 3). While 22 genes, including *Cd38*, *Cd55*, *Dntt*, *Ms4a1*, *Gfra2*, *Il7r*, and *Cxcr2*, were all downregulated in all the 3 mutant groups, the expression of *E2-Eb1*, *Il12rb2*, *Ifnar1*, *Cd4*, *Cd7*, and *IL7r* was uniquely upregulated in *TF* and *TFD* mutants compared with *DF* mutant (Figure 4C, Supplemental Figure 4A, and Supplemental Table 3). *Csf2rb* was found to be uniquely downregulated in *TF* compared with *DF* and *TFD*. Collectively, these data suggest that, with the exception of some genes, the changes in the expression of genes were similar in all 3 mutant groups.

### Dysregulated inflammatory response, metabolism, and inflammatory cytokines in HSC/Ps bearing TF, DF, and TFD mutations.

We further conducted pathway enrichment analysis of the dysregulated genes, focusing on the canonical pathways in MsigDB v6.0 between *DF*, *TF*, and *TFD* HSC/Ps versus *WT*; *TFD* versus *DF* and *TF* HSC/Ps; and *DF* versus *TF* HSC/Ps, using hypergeometric test (30). A complete list of the significantly differentially expressed pathways between these groups is provided in the Supplemental Table 5. We observed a strong overlap among the dysregulated genes between *DF*, *TF*, and *TFD* versus WT (Supplemental Table 3), suggesting that the combination of these mutations likely utilizes common oncogenic mechanisms to induce transformation. Given that *Tet2* and *Dnmt3a* are both epigenetic regulators, we speculate that the increased instability in epigenetic regulation combined with the presence of *Flt3^ITD/WT^* mutation likely facilitates the selection of a common set of dysregulated genes, which might contribute to leukemia development. To this end, we first identified pathways that were commonly up- or downregulated in *DF*, *TF*, and *TFD* gene sets versus WT (Figure 4, E and F; Supplemental Figure 4, B and C; and Supplemental Table 5). Four clusters of functional pathways were consistently upregulated in *TFD* versus WT: (a) genes involved in the inflammatory response (inflammatory cytokines, receptors, inflammasomes, and signaling by ILs), (b) genes involved in the innate immune response (TLRs, innate immune cell types, AML marker genes, and MHC class II antigen presentation), (c) genes associated with lipid metabolism (phospholipid, sphingolipid, lipoprotein, glycosphingolipid, and cholesterol metabolism), and (d) genes involved in the glycosaminoglycan metabolism pathway (hyaluronic acids, chondroitin sulfate, and other glycosaminoglycan metabolism). All 4 of these pathways are highly relevant to inflammatory responses. In contrast, the adaptive immune system (T and B cell receptor signaling pathways and ILs expressed by these cells), G-protein couple receptor (GPCR) signaling, and glycerophospholipid biosynthesis pathways were consistently downregulated in *DF*, *TF*, and *TFD* versus WT (Supplemental Table 5). We identified multiple groups of cytokines and cytokine receptors, which are involved in regulating immune and inflammatory responses. There was a consistent change in their expression in *DF*, *TF*, and *TFD* HSC/Ps versus WT. The upregulated and downregulated cytokines and receptors, which are more relevant for regulating the immune responses of the more differentiated myeloid and lymphoid cell lineages, are presented in Supplemental Figure 4A and Supplemental Table 4.

We further analyzed the pathways specifically dysregulated in *TFD* versus *DF* or *TF*, and *DF* versus *TF* mutant HSC/Ps. Our unsupervised clustering analysis showed that cases of leukemia driven by *TF* and *TFD* are more alike compared with leukemia driven by *DF* mutants. Observing the less distinct differential expression among the *TFD*, *DF*, and *TF* groups, we applied the single-sample GSEA (ssGSEA) analysis to assess the biological characteristics specific to *TFD* mutant compared with the *DF* and *TF* groups by using sample-wise pathway enrichments. We observed a set of functionally dependent inflammatory pathways that were upregulated in *TFD* versus *DF* and *TF*, including (a) intrinsic pathways, complement cascade, platelet aggregation, and coagulation pathways; (b) CXCR chemokine receptor binding and activity pathways; and (c) cell adhesion mediated by integrin and gap junction pathways. These observations suggest a more dysregulated inflammatory response of the early0phase wounding healing. It is conceivable that the formation of a chronic inflammatory condition of chronically activated complement pathway may affect the regular maturation and activation of adaptive immune response. We observed a significant downregulation of IFN-γ–mediated response and slight downregulation of the *Ifng* and *Tnf* genes in *TFD* versus *DF* and *TF* mice. A decrease in the relative proportion of CD8^+^ T cells predicted by semisupervised deconvolution of mouse data (SSMD) and a downregulation of CD8^+^ T cell markers including *Cd8a*, *Gzmb*, and *Prf1* was also seen in *TFD* versus *DF* and *TF* mice (Figure 4G and Supplemental Figure 6). It is possible these somewhat unique perturbations in *TFD* HSC/Ps contribute to more aggressive leukemia.

Next, to gain a deeper insight into the mechanisms of transformation in *TFD* mice, we interrogated additional pathways. We observed the downregulation of TCA cycle, oxidative phosphorylation, electron transport chain, NADH dehydrogenase complex, oxidative reductase, regulation of apoptosis cascade, mitochondrial calcium transporter, and other mitochondrial proteins in *TFD* versus *DF* and *TF* HSC/Ps, suggesting loss of mitochondrial functions in *TFD* versus *DF* and *TF* mice (Supplemental Figure 5). Given that the coagulation process is calcium dependent, it is possible that deprivation of calcium in an inflammatory tumor microenvironment might result in mitochondrial dysfunction observed in *TFD* mice. We also observed downregulation of lipid, steroid, and cholesterol biosynthesis in *TFD* versus *DF* and *TF* mice. The loss of mitochondrial function blocks the production of citrate, which might affect downstream acetyl-CoA and lipid biosynthesis in *TFD* mice (Supplemental Figure 5). In addition to the above-described genes and pathways, we also identified up regulation of *BCAT*-regulated genes and downregulation of AKT, NF-κB, KRAS, and EIF4E in *TFD* versus *DF* and *TF* mice. Both AKT and NF-κB are key transcriptional factors that are triggered by complement pathway in a regular wound-healing process. It is possible that the perturbation of these highly intertwined complement and coagulation pathways, including inflammation, may result in a more aggressive AML phenotype noted in *TFD* mice. Our genomic analysis revealed more common than unique sets of dysregulated pathways in HSC/Ps derived from all 3 mutant mice; this suggests a possible common oncogenic mechanism, but also group-specific functional variations that might contribute to differential manifestation of the AML phenotype.

### RNA-Seq analysis of mice bearing TF, DF, and TFD mutations show consistency with OHSU and TCGA human data of patients with AML bearing the same mutations.

**Figure 5:**
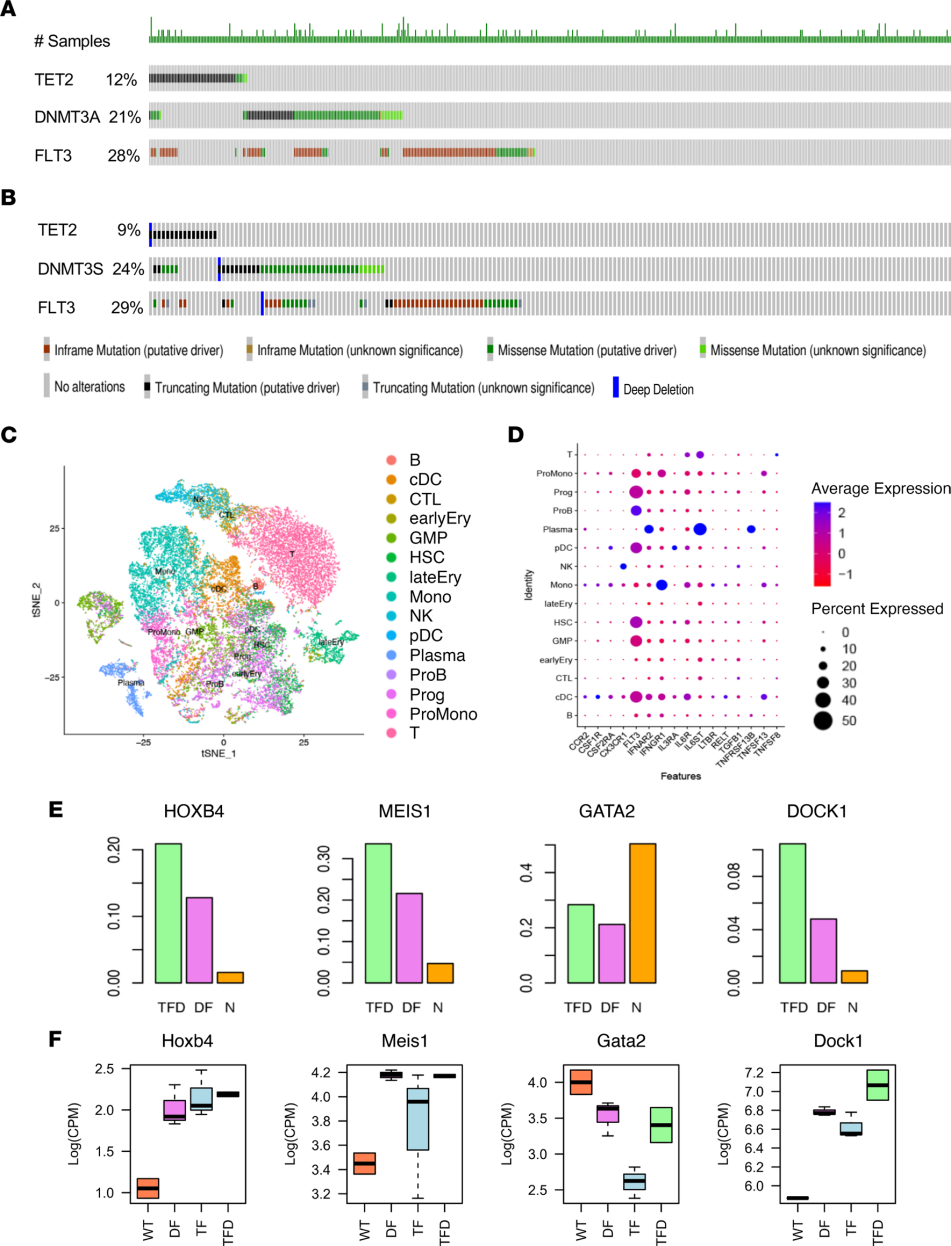
RNA-Seq data associated with *TF*, *DF*, and *TFD* were consistent with scRNA-Seq data. (**A** and **B**) OHSU and TCGA data reveal a high frequency of concurrent mutations in *DNMT3A*, *FLT3*, and *TET2* genes in patients with AML. (**C**) scRNA-Seq data from the BM of 13 human AML samples with matched genetic information to validate the cell type–specific gene expression variations inferred from the RNA-Seq data. (**D**) Cell type–specific gene expression of hematopoietic lineages. (**E**) Gene expression in scRNA-Seq data from human AML. (**F**) Gene expression from mouse RNA-Seq data.

Next, we compared the gene expression profiles of human AML with cooccurrence of the same *TFD* mutations described above in mice utilizing the OHSU and TCGA databases (Figure 5, A and B). Gene mutation and RNA-Seq profile of 199 AML patient samples was interrogated, among which the mutation rates of *TET2*, *DNMT3A*, and *FLT3* were 9%, 24%, and 29%, respectively (Figure 5B). Among the AML samples whose mutation profile and sequencing data were available, 3 (2.0%) had concurrent mutations in *TET2*, *DNMT3A*, and *FLT3^ITD^*, 8 (5.2%) showed concurrent mutations in *DNMT3A* and *FLT3^ITD^*, 10 (6.6%) had only *FLT3^ITD^* mutation, and 19 (12.6%) showed only *DNMT3A* mutation. We observed 278 and 109 upregulated, and 685 and 106 downregulated genes in the TCGA human AML *TFD* HSC/Ps versus the AML HSC/Ps, with no mutations in *TET2*, *FLT3*, and *DNMT3A*. Remarkably, the genes identified in the *TFD* AML HSC/Ps from the human TCGA data sets showed a significant overlap with the genes differentially expressed in the *TFD* HSC/Ps derived from the mutant mice versus in mice with no mutations in *Tet2*, *Flt3*, and *Dnmt3a* (*P =* 0.08 and 0.00016 for up- and downregulated genes, respectively); this suggests remarkable evolutionary conservation in the gene expression profile between mice and human AML bearing a combination of *TFD* mutations. Overall, our RNA-Seq data in *TFD* mice are consistent with the human OHSU and TCGA data, demonstrating that multiple epigenetic/genetic genes cooperate to manifest an aggressive form of AML (Figure 5, A and B).

### Cell type–specific gene expression changes associated with TF, DF, and TFD mutations.

Although the deconvolution analysis (31) could decipher cell type–specific gene expression signals from our HSC/P data, the detected cell type–specific variations need to be validated as truly cell type specific. Therefore, to further validate our HSC/P data, we utilized a single-cell RNA-Seq(scRNA-Seq) data set consisting of 30,712 cells from the BM of 13 human AML samples with identical genetic mutations, in order to validate the cell type–specific gene expression variations inferred from the tissue RNA-Seq data (32). Specifically, cell types were first inferred from the scRNA-Seq data and annotated with gene markers as shown in van Galen et al. (32), resulting in 15 cell types, including HSCs, GMPs, CMPs, megakaryocyte and erythroid progenitors (MEPs), and mature cells of the hematopoietic lineage. Differentially expressed genes in the same cell type among different genetic backgrounds were identified by using left truncated mixture Gaussian distribution–based (LTMG-based) test (33) and matched with the differentially expressed genes identified in our experimental HSC/Ps data. The analysis of the scRNA-Seq data focused on 2 specific goals: (a) validating the cell type–specific genes, especially the mutation associated genes, among different mutation groups or versus AML without the 3 mutations and (b) confirming the cell types that express the differentially expressed cytokines identified in the HSC/Ps cells (Figure 5C). Figure 5C shows the t-distributed stochastic neighbor embedding (tSNE) plots of the single cells and annotated cell types in the scRNA-Seq data. Cell type–specific genes were first identified from the scRNA-Seq data as the ones significantly upregulated in 1 cell type compared with others (*P <* 0.01 by LTMG-based test). A detailed list of cell type–specific data sets used for training of hematopoietic cell marker genes is provided in Methods and Supplemental Table 2. A significant number of genes was downregulated in the *DF*, *TF*, and *TFD* versus WT mice in a cell type–specific manner, including genes expressed in Pro-B cells (*P =* 0.03), T cells (*P =* 0.008), and erythroid progenitors (*P =* 0.0082). Importantly, the reduced expression of genes for these cell types significantly correlated with the loss of these cell types observed in mutant mice, including the *TFD* mutants. Our data also show that the cytokines that are upregulated in *DF*, *TF*, and *TFD* versus WT mice are robustly expressed by HSCs, GMPs, classical DC (cDC), and promonocytes, while the downregulated cytokines are abundantly expressed by the lymphoid lineage cells (Figure 5D). In total, 163 HSC specific genes and 197 GMP-specific genes were identified from the scRNA-Seq data, which were significantly (57 of 163, *P =* 0.02; 71 of 197, *P =* 0.008) enriched for genes that show upregulation in *DF*, *TF*, and *TFD* versus WT, which further confirmed the increased GMP numbers and frequencies observed in our primary mice HSC/Ps. We found 186 genes to be upregulated in the *DF*, *TF*, and *TFD* versus WT HSC/Ps, and these genes were also upregulated in HSCs derived from AML with *DF* or *TFD* versus nonmutated *TFD* HSCs (*P <* 1 × 10^–10^ by Fisher’s exact test), including the upregulated HSC renewal–related genes *Hoxa9*, *Hoxb3*, *Hoxb4*, and *Meis*; the hematopoietic cytokines *Ifnar2*, *Il3lra*, *Il3ra*, and *Flt3;* and the downregulated genes *Il5ra* and *Cd55*. Figure 5E shows the expression of *Hoxb4* and *Meis* in the scRNA-Seq data, and Figure 5F shows their expression in the RNA-Seq data derived from mouse HSC/Ps. We also identified *Gata2* (*P =* 0.0005) and *Dock1* (*P =* 0.04) to be consistently upregulated in *TFD* and *DF* versus *TF*, in both our mouse tissue data and the HSC single-cell data (Figure 5, E and F).

### TFD mutant–driven AML respond to a combination of drugs that target Flt3^ITD^, inflammation, and methylation.

**Figure 6:**
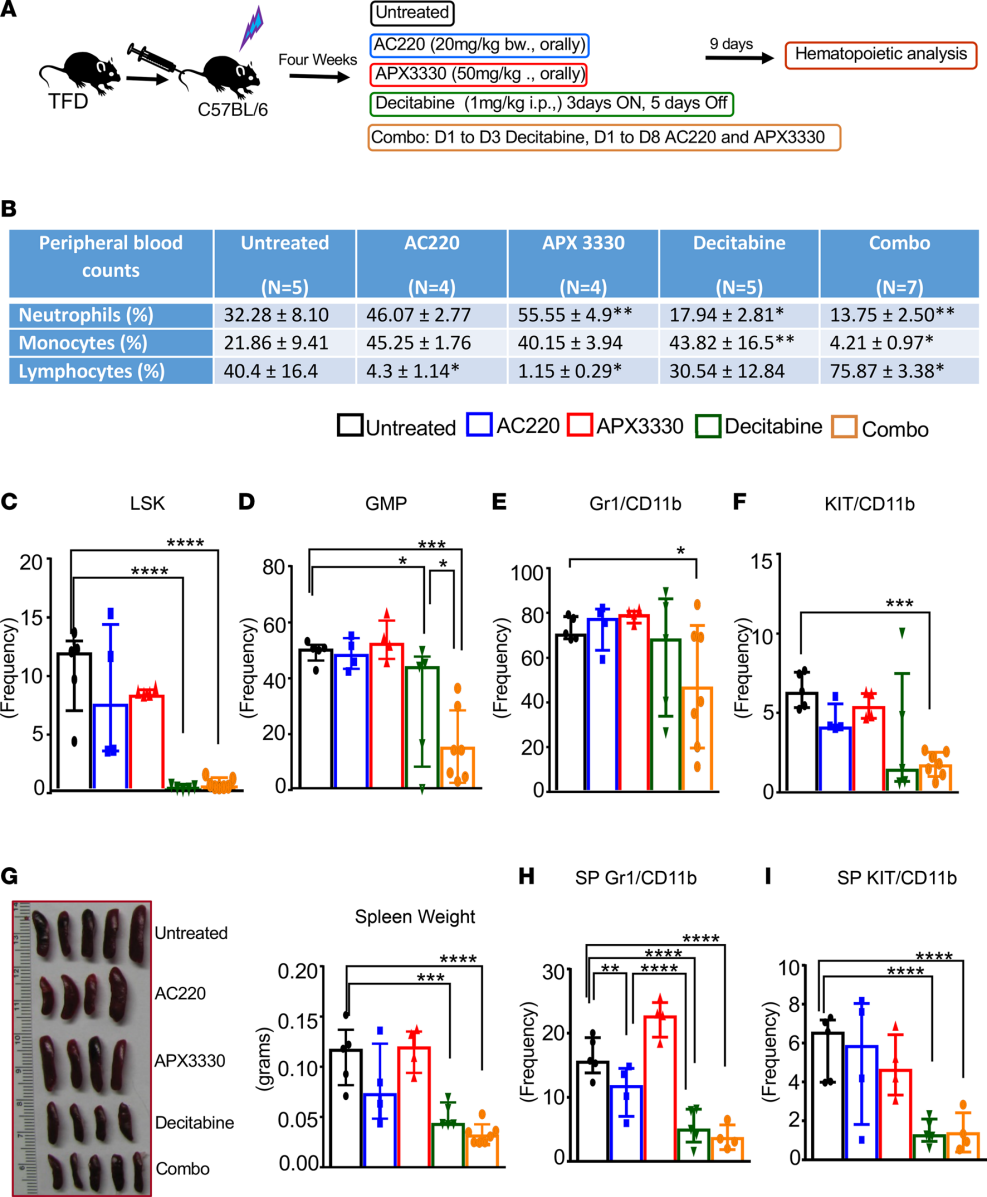
*TFD*-driven AML responds to a combination of FLT3 inhibitor, APE-1 inhibitor, and decitabine. (**A**) Schematic of the experimental design. (**B**) Table showing the effect of single and combined drug treatment on PB counts. (**C**) Frequency of LSK cells in *TFD* recipient mice treated with various drug combinations. (**D**) Frequency of GMP population. (**E**) Frequency of Gr-1/CD11b–double-positive myeloid population in BM. (**F**) Frequency of KIT/CD11b–double-positive myeloid blasts in the BM. (**G**) Representative spleen pictures and quantification data on spleen weights. (**H**) Frequency of spleen Gr-1/CD11b–double-positive cells. (**I**) Frequency of KIT/CD11b–double-positive myeloid blasts in spleen. Each data point represents a value from an individual mouse. Error bars indicate the interquartile range. Statistical analysis was performed using GraphPad version 7 using 1-way ANOVA with uncorrected Fisher’s test. *P < 0.05, **P < 0.01, ***P < 0.001, ****P < 0.0001.

Given the presence of *Flt3^ITD^* and heterozygous loss of *Tet2* and *Dnmt3a* in *TFD* mutant mice and the observed dysregulated expression of inflammatory cytokines noted in these mice, we assessed the impact of using a combination of drugs that target *Flt3^ITD^*, inflammation, and dysregulated methylation in HSC/Ps. Ref-1/APE-1 is a transcription factor activation redox regulator component of the DNA damage response in tumor cells (34). In clinical trials, the APE-1 inhibitor, APX3330, is administered orally to target APE-1 (35, 36). APX3330 has potent antiinflammatory activity via repressing the redox function of transcription factors involved in regulating inflammatory cytokines, such as Stat3 and NF-κB (37). We hypothesized that targeting inflammatory mediators, in combination with the hypomethylating agent decitabine and the *Flt3^ITD^* inhibitor AC220, might reduce *TFD*-driven leukemic burden. Briefly, 12-week-old *TFD* mice were given 5 injections of poly(I:C) on alternative days. Fourteen weeks after poly(I:C), 2 million BM cells from *TFD* mice were transplanted to lethally irradiated C57BL/6 mice. Four weeks after transplant, PB counts were assessed. A cohort of mice was randomly divided into an untreated group, an AC220-treated (20mg/kg) group, an antiinflammatory compound APX3330–treated (50mg/kg) group, a DNMT methylation inhibitor decitabine–treated (1mg/kg) group, and a group in which *TFD* mice were treated with a combination of 3 drugs. After 9 days of treatment (Figure 6A), we measured PB counts and sacrificed these mice to perform detailed hematologic analysis. As seen in Figure 6B, the major loss of lymphocytes observed in *TFD* mice was significantly restored upon treating leukemic mice with a combination of above described drugs. In addition, the frequency and the ratio of monocytes to lymphocytes in the PB was also significantly corrected in *TFD* mice treated with decitabine and more so when treated with a combination of all 3 drugs.

We next assessed the impact of this treatment regimen on the frequency of LSK cells in the BM of *TFD* mice. As seen in Figure 6C, the profound increase in the LSK cell frequency observed in *TFD* mice was corrected upon treating the mice with decitabine or with the combination of all 3 drugs. Importantly, the expansion of GMPs seen in the BM of *TFD* mice was rescued in response to 3-drug combination treatment (Figure 6D). Likewise, the frequency of terminally differentiated Gr-1/CD11b–double-positive cells, as well as KIT/CD11b–double-positive myeloid blasts, was also reduced upon treating the mice with the 3-drug combination (Figure 6, E and F). The most profound impact was noted in the spleen size and weight (Figure 6G). The significant increase in spleen size noted in *TFD* mice was completely rescued in decitabine and the 3-drug combination–treated *TFD* mice. The frequency of Gr-1/CD11b–double-positive cells, as well as KIT/CD11b–double-positive myeloid blasts in the spleen, was also rescued in decitabine and combination treatment groups (Figure 6, G–I). Interestingly, the combined drug treatment uniquely impacted Gr-1/CD11b–double-positive cells in BM and CD11b^+^ cells in PB (Supplemental Figure 5A), and it increased the frequency of CD3^+^ lymphoid cells in BM (Supplemental Figure 5B). These observations were consistent with the rescue of PB counts seen in Figure 6B. Taken together, *TFD*-driven aggressive AML responds to a combination therapy that targets the mutated receptor tyrosine kinase, epigenetic changes due to *Tet2* and *Dnmt3a* mutations, and inflammation caused by the presence of genetic and epigenetic mutations.

### Patient-derived multimutational AML cells respond to combinational drug treatment in a patient-derived xenograft (PDX) model.

**Figure 7:**
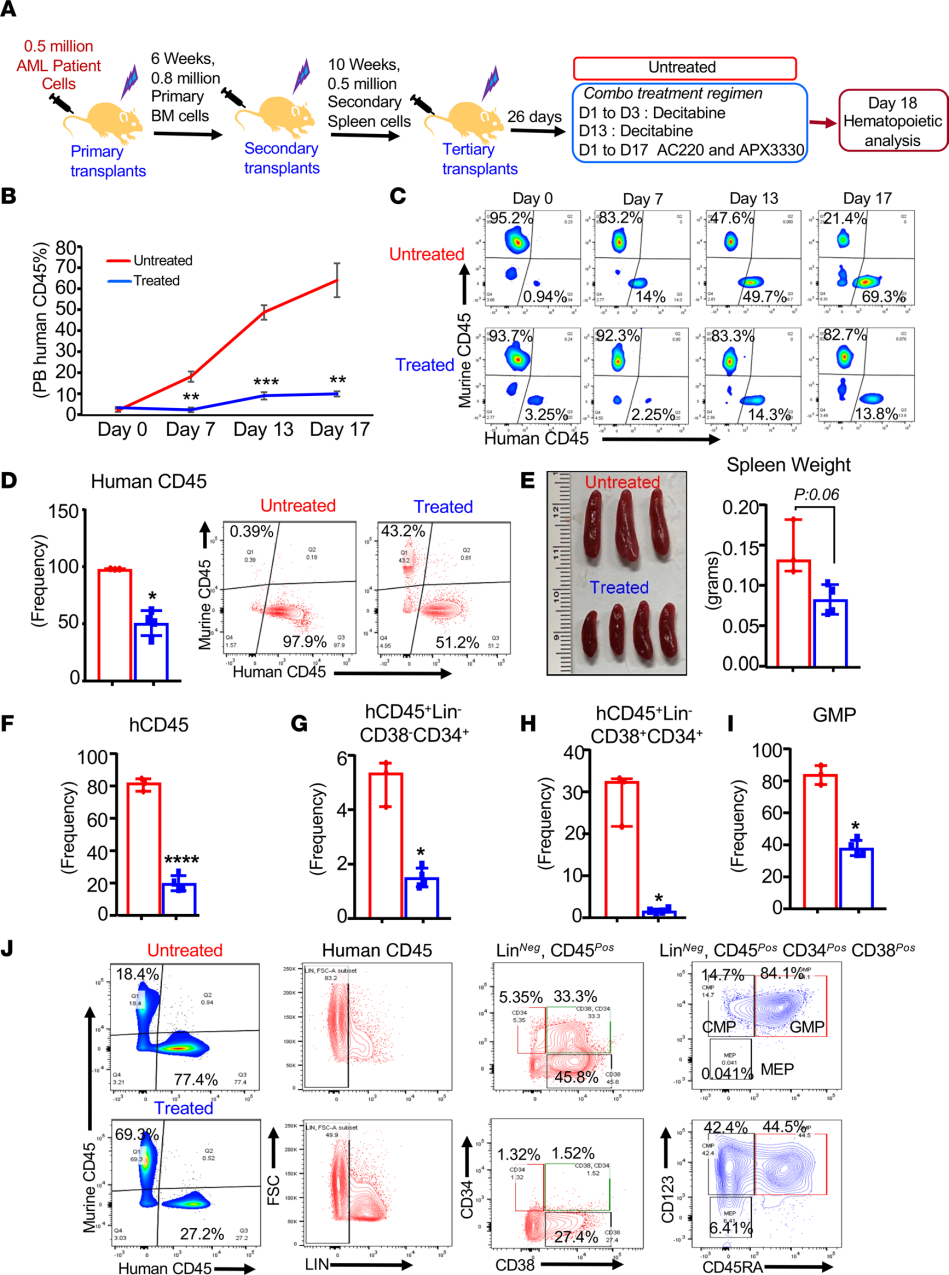
Multimutational AML PDX responds to combinational drug treatment. (**A**) Schematic of experimental design. (**B**) PB human CD45 engraftment was monitored at indicated time points. (**C**) Representative flow profiles of human CD45^+^ cells on indicated days after drug treatment. After 17 days of treatment, all these mice were sacrificed to perform detailed hematopoietic analysis. (**D**) Quantification data of the frequency of human CD45^+^ cells in the BM and representative flow profiles. (**E**) Representative spleen pictures and quantification data of spleen weights. (**F**) Frequency of human CD45^+^ cells in the spleen. (**G**) Frequency of human CD45^+^Lin^–^CD38^–^CD34^+^ leukemic stem cells in the spleen. (**H**) Frequency of human CD45^+^Lin^–^ CD34^+^ CD38^+^–double-positive cells in the spleen. (**I**) Frequency of GMPs (human CD45^+^Lin^–^CD34^+^ CD38^+^CD45RA^+^CD123^+^) population in the spleen. (**J**) Representative flow profiles of human CD45^+^ leukemic stem cells and progenitors from the spleen of control and treated groups. Error bars indicate the interquartile range.

We wanted to validate the combinatorial drug regimen in PDX model. We utilized a BM sample derived from a patient with multimutational AML bearing *FLT3^ITD^* (*ins46*), *DNMT3A-R882H*, *NPM1-W288FS12*, and *CHEK2* mutations and transplanted these cells into sublethally irradiated NSGS mice. These cells were expanded twice in NSGS mice. Two weeks after tertiary transplantation, human cell engraftment in PB was assessed by staining PB cells with human CD45 antibody. These mice were randomly divided into vehicle-treated and drug-treated groups. The drug-treated group received a combination of FLT3 inhibitor AC220 (20 mg/kg, orally), antiinflammatory compound APX 3330 (50 mg/kg, orally), and DNMT methylation inhibitor decitabine (1 mg/kg, i.p.) as showed in the experimental design presented in Figure 7A. As shown in Figure 7B, a combination drug treatment significantly inhibited the PB engraftment of human AML CD45^+^ cells over an 18-day period (red versus blue line). Representative flow profiles in Figure 7C show the consistent reduction in the engraftment of human CD45^+^ cells in the PB of combined drug–treated mice compared with the untreated group. After 18 days of drug treatment, we sacrificed the mice to assess the impact of drug treatment on the BM of AML-bearing mice. As seen in Figure 7D and consistent with our observations in the PB of drug-treated mice, the engraftment of drug-treated mice in the BM was significantly reduced compared with the vehicle-treated group. Likewise, we observed a reduction in splenomegaly in mice treated with the 3-drug combination (Figure 7E).

We next assessed the effect of 3-drug treatment on leukemic stem and progenitor cells in the BM and spleen. Consistent with the decrease in the frequency of human CD45^+^ cells, a 3.35-fold decrease in the frequency of leukemic stem cells (Lin^–^CD38^–^CD34^+^) was also observed in the spleen of drug-treated mice compared with controls (Figure 7, F and G). Likewise, the frequency of Lin^–^CD38^+^CD34^+^–double-positive cells was also reduced compared with controls (Figure 7H). The frequency of myeloid progenitors (GMP) was also reduced in drug-treated mice compared with controls (Figure 7I). Representative flow profiles of control and drug-treated mice are presented in Figure 7J. Next, we assessed the impact of combined drug treatment on human mature myeloid (human CD45^+^CD33^+^CD14^+^) cells in the BM, spleen, and PB. Combined drug treatment showed a significant reduction in the frequency of mature myeloid cells in these tissues compared with controls (Supplemental Figure 7). Overall, these data demonstrate that both mouse and human AML cells respond efficiently to repress the growth of leukemic cells in response to the 3-drug combination treatment. We acknowledge the fact that the human AML sample used in our study doesn’t precisely carry all the 3 mutations described in the mouse model. We were unable to secure a human AML sample carrying the exact TFD mutations.

## Discussion

Whole exome sequencing and next-generation sequencing in patients demonstrate cooccurrence of *FLT3*^ITD^, *TET2*, and *DNMT3A* mutations in patients with lymphoid and myeloid malignancies; however, it is unclear how these mutations cooperate and contribute to transformation and poor overall survival. Although homozygous double-mutant AML genetic mouse models of *Tet2^–/–^ Flt3^ITD/ITD^* (1, 5) and *Dnmt3a^fl/fl^ Cre Flt3^ITD/ITD^* (2) have been shown to promote leukemogenesis, heterozygosity of these mutations and their role in driving leukemogenesis in a head-to-head manner has never been examined, to our knowledge. It is critical to examine the role of heterozygous mutations in driving AML, as most patients possess heterozygous mutations of *TET2*, *DNMT3A*, and *FLT3^ITD^*. Recently, triple-mutants of *NPM1–FLT3-ITD–IDH1/2* (38), *WT1–FLT3-ITD–NUP98-NSD1* (39) fusion, and *DNMT3A–NPM1–FLT3-ITD* (40) have been reported in patients with AML with poor overall survival. In the present study, we have generated mice expressing heterozygous *Flt3*^ITD^ driven by its endogenous promoter and concomitant for either heterozygous loss of *Tet2* or *Dnmt3a* or both *Tet2* and *Dnmt3a* to assess the biological consequences of coexistence of these mutations. We show that the effect of combination of 2 or 3 mutations on a hematopoietic phenotype in HSC/Ps is qualitatively similar; however, the effect is significantly greater in *TFD* mice, followed by *DF* and then *TF* mice. The disease manifestation in the primary transgenic mutant mice, as well as after transplantation of mutant cells, is very similar between *TF*, *DF*, and *TDF* mutants; however, the severity and latency of the disease is significantly greater in the setting of triple heterozygosity, suggesting a cooperative effect of these 3 mutations in driving AML.

We applied RNA-Seq data to dissect the molecular mechanisms of cooperation between mutations in a signaling molecule and epigenetic modifiers by conducting a head-to-head comparison between the double-heterozygous mutants *TF* and *DF* and a triple-heterozygous mutant *TFD*. Pathway enrichment analysis revealed that the HSC self-renewal genes, including *Hoxa9*, *Hoxa3*, *Hoxa7*, *Etv6*, and *Gata2*, were differentially expressed in all 3 mutant groups, suggesting that these genes may function as convergent nodes for cooperative transformation, which requires the combination of these mutations. Our data on upregulation of *Hoxa9*, *Hoxb3*, and downregulation of *Gata2* are consistent with earlier studies shown in homozygous *Tet2^–/–^/Flt3^ITD^* and *Dnmt3a^–/–^/Flt3^ITD^* double-mutant mouse models (1, 2). Our pathway analysis revealed a common set of dysregulated pathways in double- and triple-heterozygous mutants, suggesting possible common oncogenic mechanisms utilized by *Tet2* and *Dnmt3a*, in combination with *Flt3^ITD^*.

Furthermore, our pathway enrichment analysis revealed that the upregulated HSC self-renewal genes are common between *TF* and *DF* double mutants, and some are common between double and triple mutants. Among the HSC self-renewal genes that are common between the double and triple mutants, *Myc1*, *Zfp521*, *Meis1*, and *Zfp532* have been found to be functional not only in HSC self-renewal and differentiation, but also in leukemia progression and maintenance. These may contribute to *TFD* aggressiveness compared with *DF* and *TF*. Zinc finger protein genes *Zfp521* was upregulated in both *TF* and *TFD*, but not in *DF*. Likewise, *Zfp532* was found to be upregulated both in *DF* and *TFD* but not in *TF* mutants, suggesting that differential upregulation of zinc finger protein genes in *TF-* and *DF*-driven leukemias. Quantitatively, either one of the zinc finger protein genes was upregulated in *TF* and *DF*, while both *Zfp521* and *Zfp532* were uniquely upregulated in *TFD* HSC/Ps, possibly contributing to aggressiveness of *TFD*-driven leukemia.

We identified multiple groups of cytokines that are involved in immune and inflammatory response. Our RNA-Seq data on cytokines identified in experimental mouse data sets are consistent with scRNA-Seq data on cell type–specific expression of cytokines identified in patients with AML with triple-heterozygous mutations. Cytokine and receptor expression seen in our mouse data set, including *Il6st*, *Ccr2*, *Csf1r*, *Ifngr1*, *Ltbr*, *Il3ra*, *Relt*, *Tnfsf13*, *Ifnar2*, *Csf2ra*, *Tnfrsf13b*, *Il6ra*, *Tnfsf8*, *Flt3*, and *Tgfb*, correlated with the expression seen in scRNA human AML scRNA-Seq data. These data suggest that triple-mutation genes cooperate to manifest an aggressive form of AML by expressing a similar set of cytokines in murine and human *TFD*–driven leukemia. Patients with AML manifest increased coagulation compared with healthy individuals (41). In the current study, our ssGSEA analysis revealed greater coagulation activity, as well as elevated intrinsic pathway and CXCR chemokine binding in *TFD* compared with *DF* and *TF* mutant HSC/Ps, suggesting that a dysregulated inflammatory response might contribute to hypercoagulation and aggressiveness in *TFD* mutants.

## Methods

### Experimental mice.

Mice were housed in pathogen-free conditions at the Indiana University Laboratory Animal Research Center. *Flt3^ITD/WT^* (42) mice were crossed with *Tet2^–/–^* (43) mice to generate double mutants of *Tet2^+/–^ Flt3^ITD/WT^* (*TF*) mice. *Flt3^ITD/WT^* mice were crossed with *Dnmt3a*^+/fl^ MxCre (44) mice to generate double mutants of *Flt3^ITD/WT^ Dnmt3a^+/–^ Cre* positive mice (*DF*). To generate triple-heterozygous compound mutants of *Tet2^+/–^ Flt3^ITD/WT^ Dnmt3a^+/–^* (*TFD*), double mutants of *Tet2^+/–^ Flt3^ITD/WT^* were crossed with *Dnmt3a*^+/fl^ MxCre mice. WT (C57BL/6) mice were procured from the In Vitro Core facility at Indiana University. All the experimental animals, including WT mice, were treated with poly(I:C) at 8–10 weeks of age and analyzed for hematopoietic phenotype after 8–9 weeks of poly(I:C) injection.

### Preparation of single-cell suspension and flow cytometry analysis.

Immunophenotyping was performed as described previously (45). A single-cell suspension of BM and spleen was prepared. Briefly, BM was flushed with IMDM (Invitrogen), RBCs in the BM were lysed using the ammonium chloride RBC lysis buffer, and cells were resuspended in PBS containing 0.2% bovine serum albumin (MilliporeSigma) and 10% rat serum (MilliporeSigma). An equal number of cells was used for staining with flow antibodies. Spleens were crushed between the microscopic slides, and single-cell suspension were filtered through a 50 μm nylon filter followed by RBC lysis similar to BM. Multiparameter analysis of stained single cells was subjected to flow cytometric analysis using 5-laser LSRII with diva software (BD biosciences), and the data were analyzed using FlowJo software. A complete list of flow antibodies and reagents is provided in Supplemental Table 1.

### BM transplantation.

All the experimental animals including WT mice were treated with poly(I:C) at 8–10 weeks of age and transplanted after 8–9 weeks of poly(I:C) injection. Two million whole BM cells from WT, single heterozygous for *Tet2^+/–^*, *Flt3^ITD/WT^*, and *Dnmt3a^+/–^*; double heterozygous for *Tet2^+/–^ Dnmt3a^+/–^* (*TD*), *Tet2^+/–^ Flt3^ITD/WT^*(*TF*), and *Dnmt3a^+/–^ Flt3^ITD/WT^* (*DF*)*;* and triple heterozygous for *Tet2^+/–^ Flt3^ITD/WT^ Dnmt3a^+/–^* (*TFD*) were injected into lethally irradiated C57BL/6 mice through tail vein injection and monitored for disease progression. When moribund, mice were analyzed for hematopoietic characterization.

### RNA isolation.

RNA was extracted from whole BM cells harvested from bones of WT, *TF*, *DF*, and *TDF* mice washed twice with PBS. RNA was collected using Trizol reagent (15596026, Ambion); they were then treated with DNase using DNase supplied in TURBO DNA-free Kit (AM1907, Ambion). The obtained RNA was repurified using RNeasy kit (74106, Qiagen) and sequenced at the Center for Medical Genomics, Indiana University School of Medicine, as described earlier (46).

### RNA-Seq processing and analysis.

The sequencing data were next assessed using FastQC (Babraham Bioinfomatics) and then mapped to the mouse genome (UCSC mm10) using STAR RNA-Seq aligner (47) with the parameter: “—out SAM mapq UNIQUE 60”. Uniquely mapped sequencing reads were assigned to mm10 refGene genes using featureCounts (48). Genes with read CPM > 0.5 in more than 3 of the samples were kept. The data were further normalized using reads per kilobase million (RPKM). Differential gene expression analysis was conducted by using the DESeq2 method with an FDR less than 0.05 as the significant cutoff (49). Pathway enrichment analyses was conducted by hypergeometric tests against mouse gene ontology (GO) and selected hematopoietic system–related gene sets, with *P* value less than 0.01 as the significant cutoff (30). Mice raw data were approved by NCBI GEO database with accession no. GSE182859.

### Identification and characterization of hematopoietic system– and AML-related gene sets.

We collected hematopoietic cell–specific expressed genes and functional marker genes from 3 different sources, including (a) direct utilization of mouse GO, (b) gene sets collected from literature, and (c) marker genes trained from independent bulk cell data. Specifically, for the marker genes, we trained 2,877 genes of 14 cell types including hematopoietic stem cells, common lymphoid progenitors, granulocyte-macrophage progenitors, megakaryocyte lineage-committed progenitors, erythroid cells, megakaryocyte-erythrocyte progenitors, multipotent progenitors, early myeloid progenitors, mature myeloid cells, precolony forming unit erythroid cells, premegakaryocytic/erythroid progenitors, B cells, and CD4^+^ and CD8^+^ T cells from 2 microarray bulk cell data sets — GSE14833 and GSE27787 — collected from the GEO database (Supplemental Table 2).

### Pathway enrichment analysis.

This analysis was conducted by using a hypergeometric test against the collected hematopoietic pathways and canonical pathways collected from MsigDB v6.0 (31)

### Hematopoietic deconvolution analysis.

We applied our in-house–developed mouse data deconvolution method, namely SSMD. Detailed computational approaches used for the deconvolution analysis are as described in Lu et al. (31). Pearson correlation was used to evaluate the association between flow cytometry–identified and gene expression–predicted cell proportions, which support the change of hematopoietic cell lineages with different mutation combinations.

### Public scRNA-Seq data analysis.

To validate our findings in an AML mouse model with human AML, we retrieved high-quality scRNA-Seq data, GSE116256, from the GEO database. The data set contains scRNA-Seq profiles of BM collected from 35 patients with AML. We excluded AML samples that harbored other mutations commonly seen in AML and selected 1 sample bearing a combination of *DNMT3A*, *FLT3*, and *TET2* mutation (AML210A), 3 samples bearing *DNMT3A* and *FLT3* mutations (AML419A, AML997, and AML328), 2 samples bearing *DNMT3A* mutations (AML210A and AML475), 1 sample bearing *DNMT3A* and *TET2* mutations (AML556), 1 sample bearing *FLT3* mutation (AML329), and 4 samples lacking mutations in *DNMT3A*, *FLT3*, or *TET2* (AML1012, AML916, AML371, and AML870) to validate the results identified using RNA-Seq data from our mouse mutants. Differential expression analysis was conducted by using a left truncated mixture Gaussian model, with *P <* 0.01 as the significance cutoff

### Statistics.

Each data point represents a value from an individual mouse in their respective groups. Data are shown as mean ± SEM. Statistical analysis was performed using GraphPad version7 by 1-way ANOVA with uncorrected Fisher’s test.

### Study approval.

All studies were approved by Indiana University Laboratory Animal Resource Center. All animals were maintained in pathogen-free facility at Indiana University School of Medicine, Indianapolis. All animal procedures were conducted in accordance with the *Guide for the Care and Use of Laboratory Animals* (National Academies Press, 2011) and were approved by the IACUC at Indiana University School of Medicine. Written informed consent was given by patients for collecting samples. Ethical approval for collecting patient samples was approved (protocol no. 1011003088) by Indiana University.

## Author contributions

BR and RK conceptualized the study, designed the experiment, analyzed the data, and wrote the manuscript. RSM initiated and conceptualized the study, designed the experiments, and analyzed the data. PLR, SKP, and JZ assisted with experiments and provided scientific inputs. MRK provided the APX3330 reagent. SP and CZ conceptualized the study. CZ performed RNA-Seq analysis and wrote the manuscript.

## Supplementary Material

Supplemental data

Supplemental table 5

## Version 1 09/08/2022

Electronic publication

## Version 2 09/09/2022

The references were renumbered and additional corresponding authors were added
