# Supplementary material for: Combined heterozygosity of FLT3ITD, TET2, and DNMT3A results in aggressive leukemia
Source: JCI Insight. 2022 Sep 8;7(17):e162016. doi: 10.1172/jci.insight.162016 (PMC9536269; doi:10.1172/jci.insight.162016)
Supplement: Supplemental data [file jciinsight-7-162016-s229.pdf]

Supplementary Material

Combined heterozygosity of *FLT3*^{ITD}, *TET2* and *DNMT3A* results in aggressive leukemia

Baskar Ramdas[1*,], Palam Lakshmi Reddy[1], Raghuveer Singh Mali[1], Santhosh Kumar Pasupuleti[1], Ji Zhang[1], Mark R. Kelley[1], Sophie Paczesny[2*], Chi Zhang[3*] and Reuben Kapur[1,3,4,5*]

Supplementary Figure 1

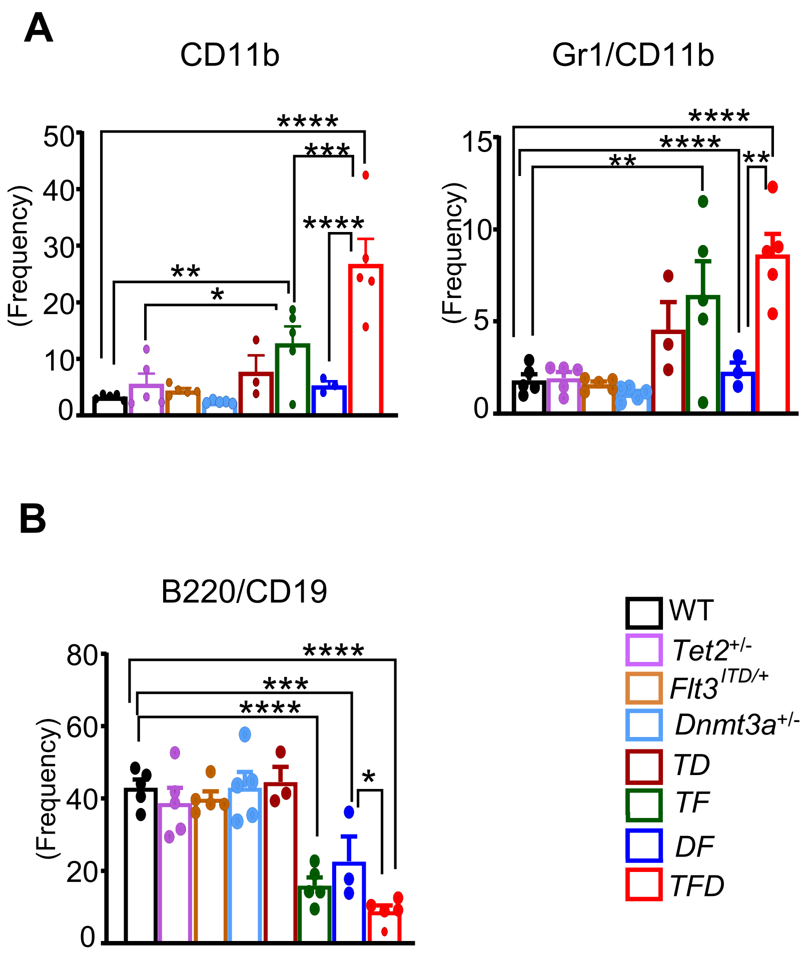

Supplementary Figure 1. Accumulation of immature myeloid cells in the spleen of *TFD* mice. WT, *Tet2*$^{+/-}$, *Flt3*$^{ITD/WT}$, *Dnmt3a*$^{+/-}$, *TD, TF, DF and TFD* mice were treated with poly (I:C) as described in methods and after 2 months poly (I:C) treatment harvested spleen and Immunophenotyping of myeloid and B cells was performed using various cell surface markers by flow cytometry. **(A)** Frequency of CD11b and Gr1/CD11b cells. **(B)** Frequency of B220/CD19 cells. Each data point represents value from individual mice. Error bars indicate the standard error of mean. Statistical analysis was performed using graph pad version 7 using Annova one way with uncorrected Fisher`s test. *p<0.05, **p<0.01, ***p<0.001, ****p<0.0001.

Supplementary Figure 2

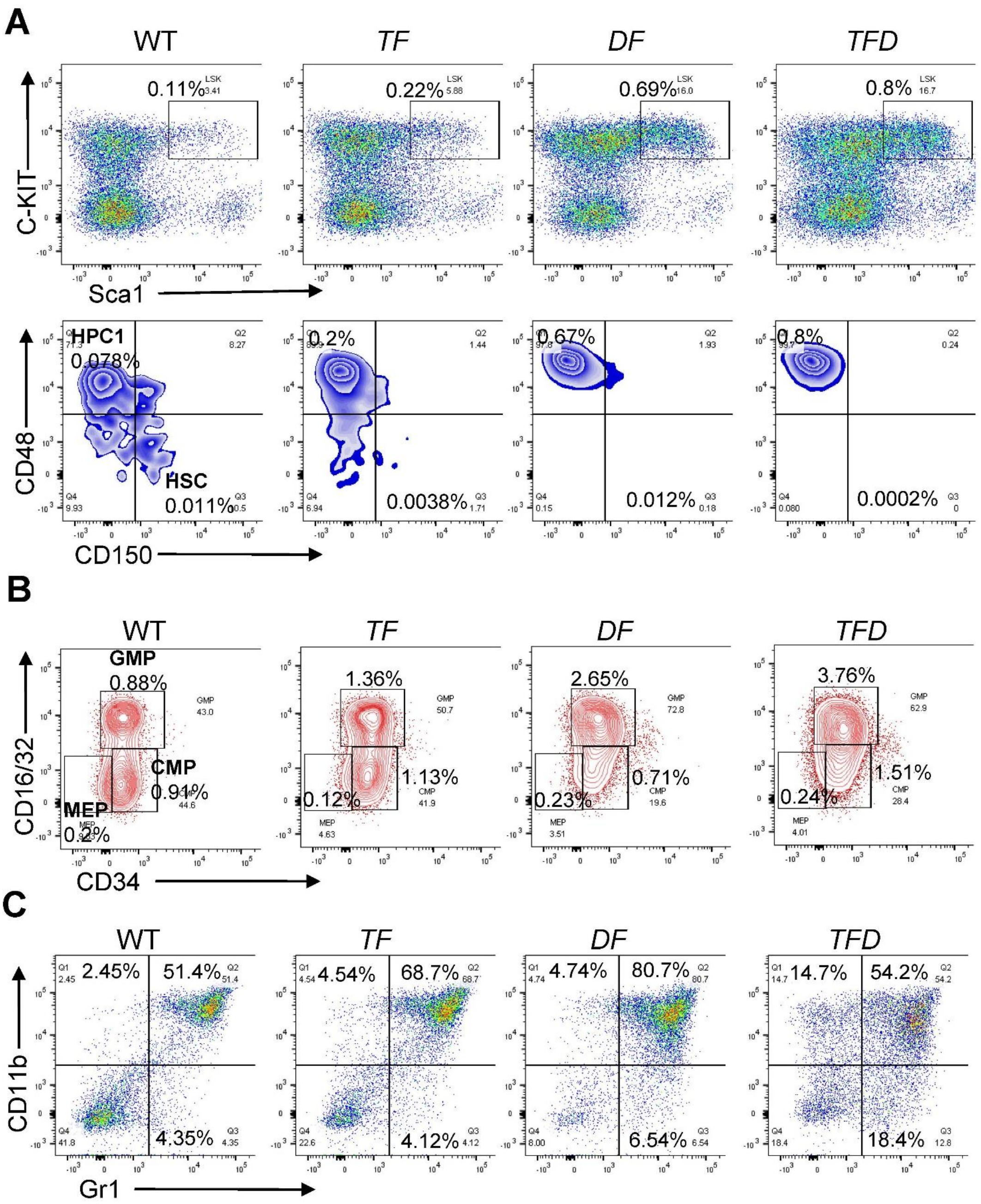

Supplementary Figure 2 Combined heterozygous loss of *Tet2* and *Dnmt3a* and expression of *Flt3*$^{ITD/WT}$ causes transplantable myeloid leukemia in mice.

Bone marrow cells (2 x 10^6 cells) from WT, *Tet2*$^{+/-}$:*Flt3*$^{ITD/WT}$, *Dnmt3a*$^{+/-}$:*Flt3*$^{ITD/WT}$, and *Tet2*$^{+/-}$:*Dnmt3a*$^{+/-}$:*Flt3*$^{ITD/WT}$ mice were transplanted into lethally irradiated C57BL/6 mice through tail vein and monitored for disease progression Mice were analysis on moribund. **(A)** Upper panel shows the representative flow prolife of LSK cells and lower panel shows the representative flow prolife of HSC population of indicated genotype recipients **(B)** Representative flow profile of committed progenitors of indicated genotype recipients **(C)** Representative flow prolife of Gr1/CD11b terminally differentiated myeloid cells.

Supplementary Figure 3

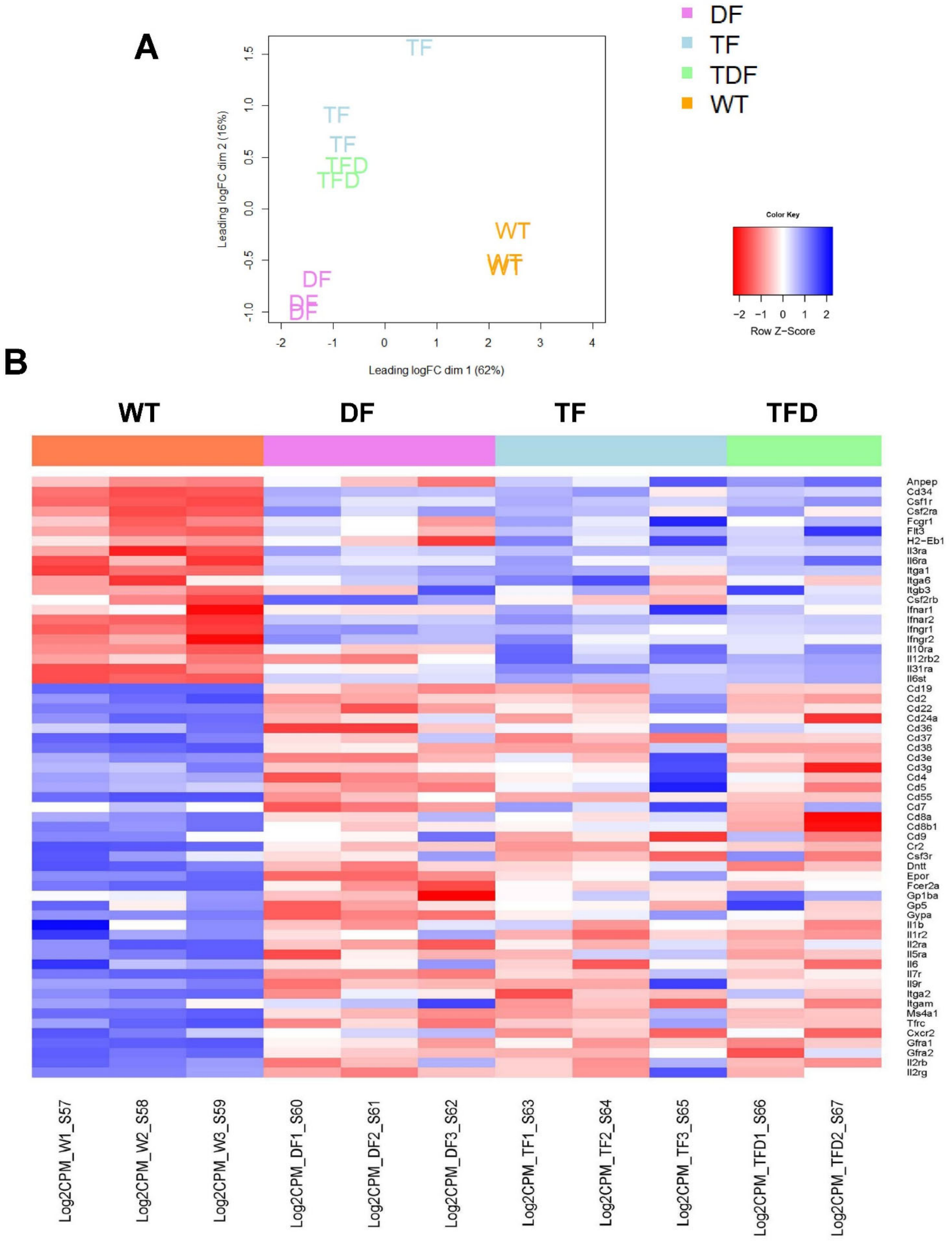

Supplementary Figure3. RNASeq analysis of bone marrow cells derived from *TF, DF* and *TFD* mice. **(A)** MDS plot made by whole transcriptomics profile reflects distinct differences between WT and *TF*, *DF and TFD* groups. **(B)** Heat map showing gene expression profile of dysregulated hematopoietic cytokine genes in *DF, TF, TFD* vs WT.

Supplementary Figure 4

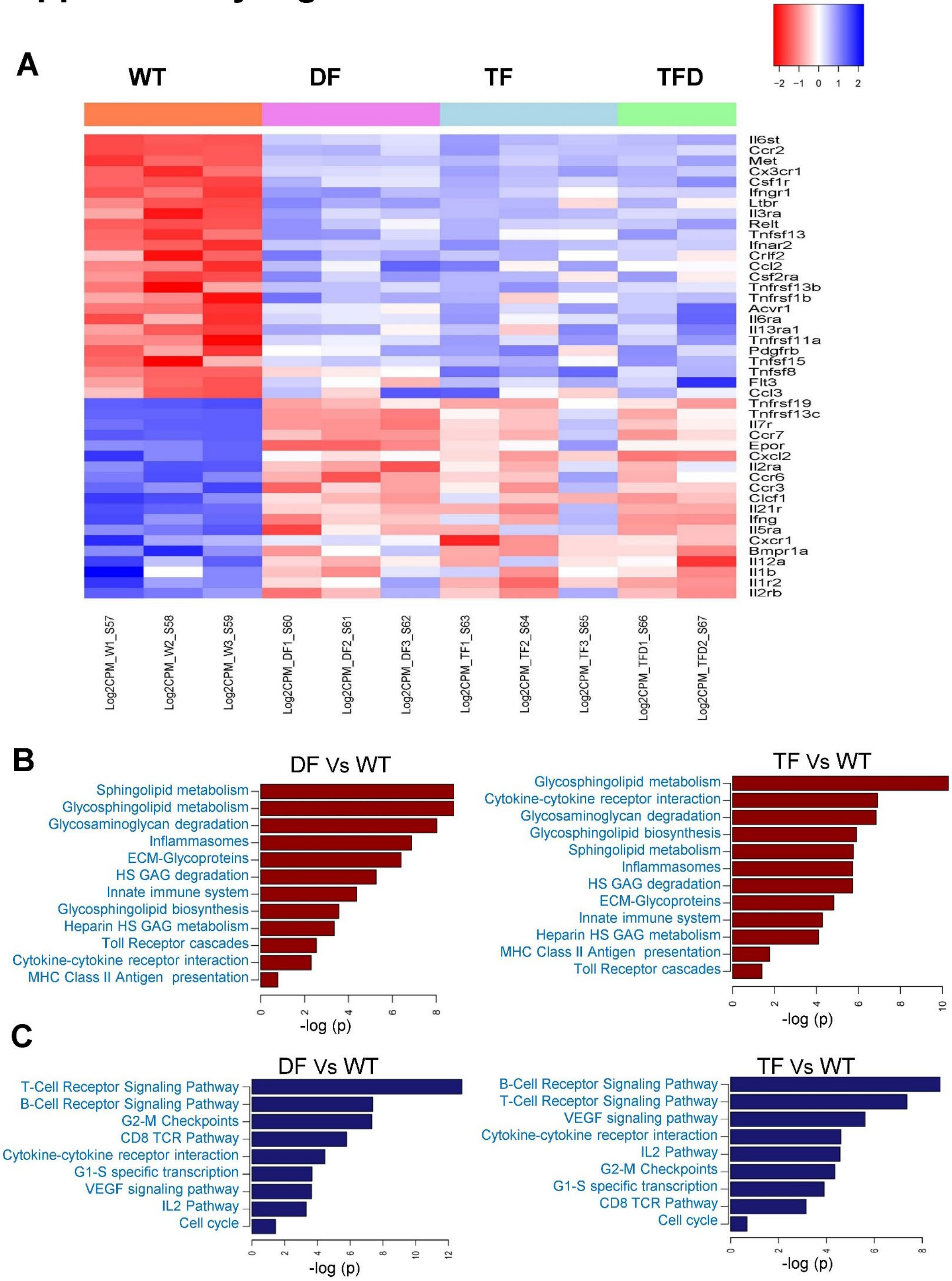

Supplementary Figure 4. RNASeq analysis of bone marrow cells derived from *TF, DF* and *TFD* mice. **(A)** Heat map showing gene expression profile of dysregulated inflammatory cytokine genes in mutants vs WT **(B)** Pathway enrichment analysis of upregulated genes in double mutants *DF* and *TF* relative to WT **(C)** Pathway enrichment analysis of downregulated genes in double mutants *DF* and *TF* relative to WT mice.

Supplementary Figure 5

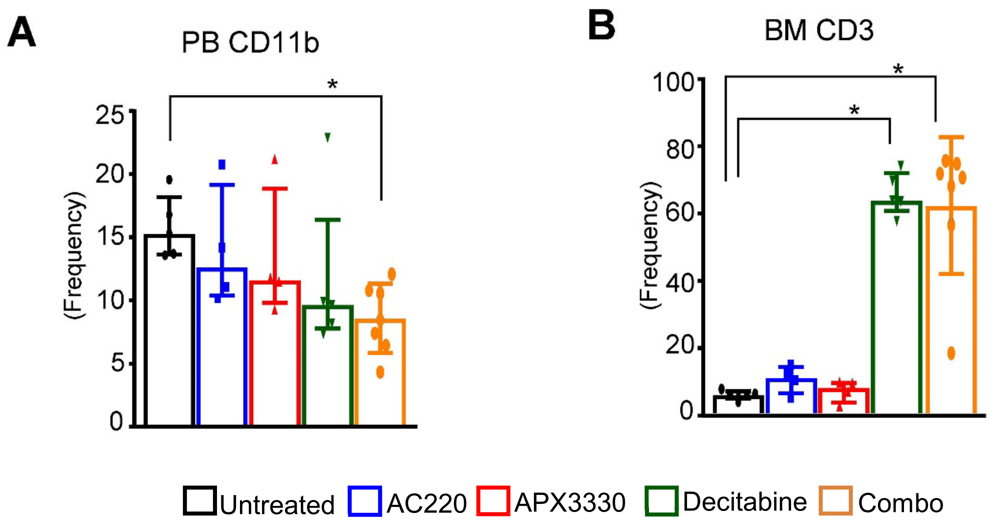

Supplementary Figure 5. *TFD* driven AML responds to a combination of Flt3 inhibitor, APE1 inhibitor and decitabine. (A) Effect of drug treatment on the frequency of PB CD11b single positive myeloid cells and **(B)** CD3 positive lymphoid cells in the bone marrow.

Supplementary Figure 6

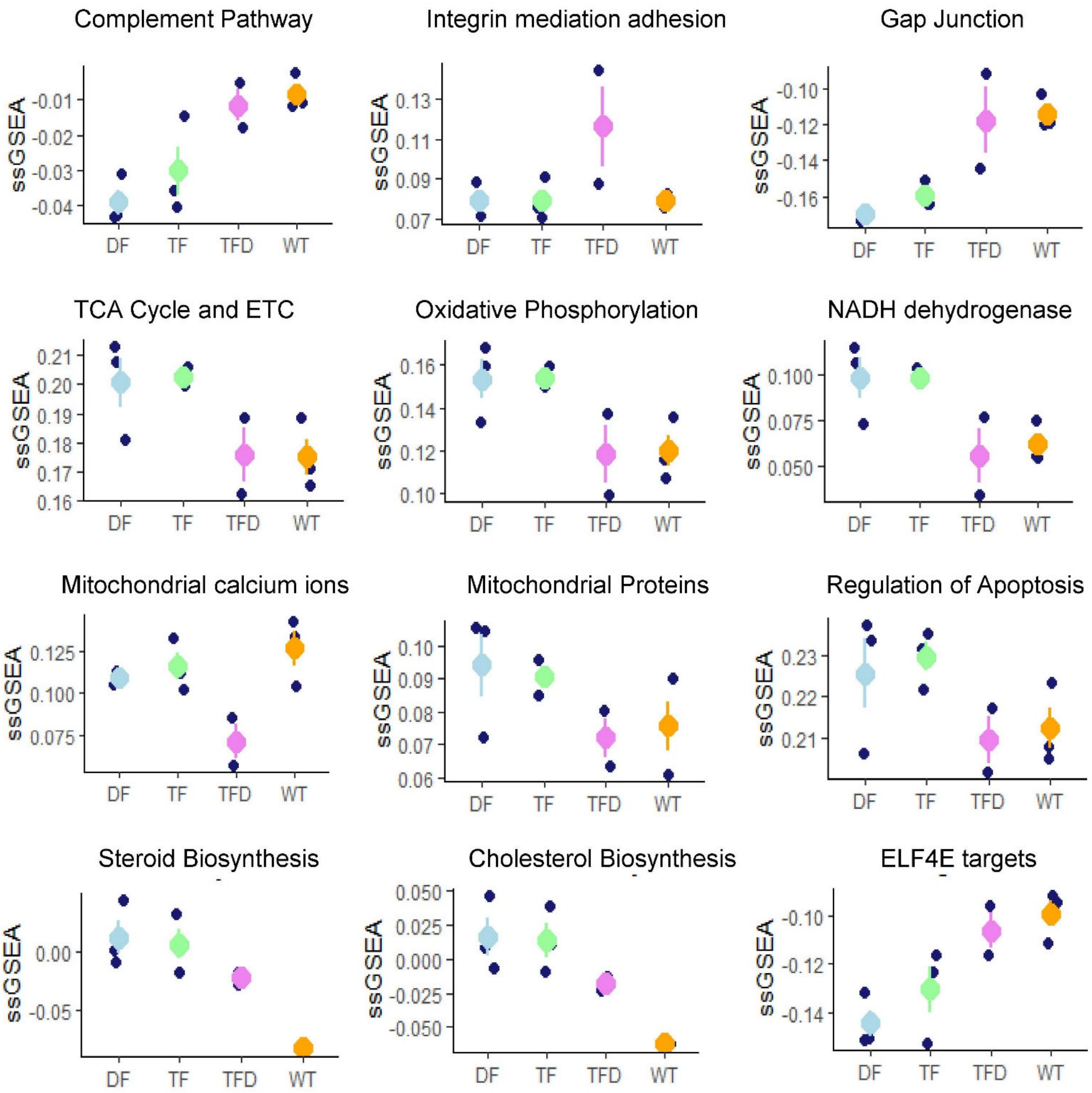

Supplementary Figure 6. Single sample GSEA analysis specific to *TFD* mutant compared to *DF* and *TF* using sample wise pathway analysis. Interrogation of additional pathways revealed the dysregulation of TCA cycle, oxidative phosphorylation, electron transport chain, NADH dehydrogenase complex, oxidative reductase, regulation of apoptosis cascade, mitochondrial calcium transporter, and other mitochondrial proteins

Supplementary Figure 7

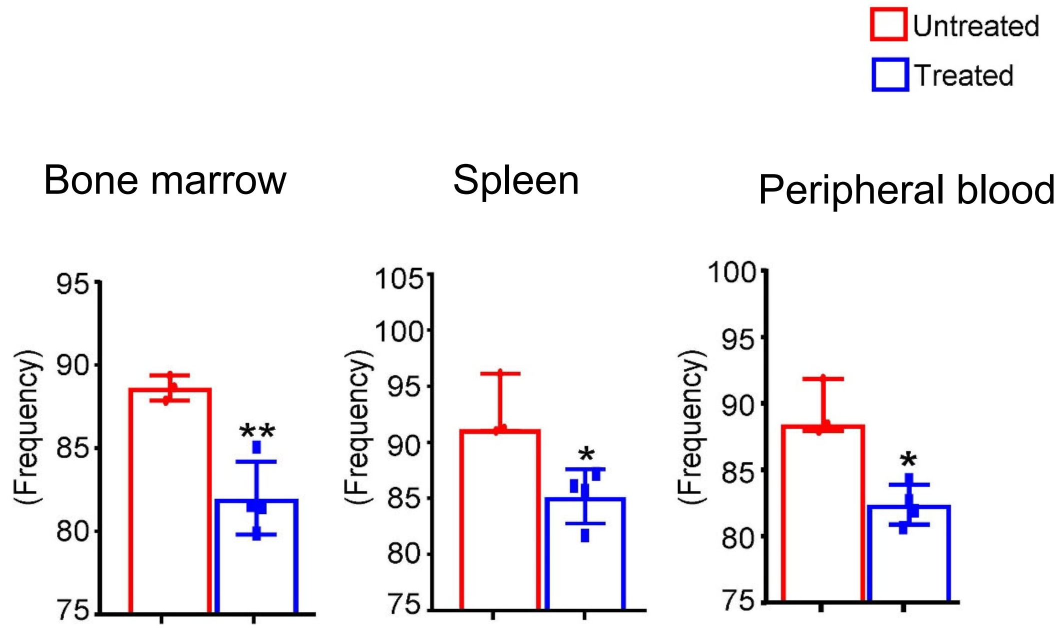

Supplementary Figure 7. Multi-mutational AML patient derived xenograft responds to combinational drug treatment

Frequency of myeloid lineage monocyte cells (human $CD45^{+}$, $CD33^{+}$, $CD14^{+}$) from Bone marrow, Spleen and Peripheral blood.

Supplementary Table 1 List of reagents

REAGENT or RESOURCE	SOURCE	IDENTIFIER
Murine Flow Antibodies		
Ter119 PE	Biolegend	Cat # 116208
B220 PE	Biolegend	Cat # 103208
Gr1 PE	Biolegend	Cat # 108408
CD11b PE	Biolegend	Cat # 101208
CD3 PE	Biolegend	Cat # 100308
C-KIT APC	Biolegend	Cat # 105812
Sca1 PE Cy7	Biolegend	Cat # 122514
CD48 APC Cy7	Biolegend	Cat # 103432
CD150 PerCPCy5.5	Biolegend	Cat # 115922
Sca1 PerCPCy5.5	Biolegend	Cat # 122524
CD16/32 PE Cy7	Biolegend	Cat # 101318
CD34 BV421	BD Biosciences	Cat # 562608
CD71 APC	Biolegend	Cat # 113820
CD19 APC	ebiosciences	Cat # 17-0193-80
CD11b APC	Biolegend	Cat # 101212
C-KIT APC Cy7	Biolegend	Cat # 105826
CD45 APC	Biolegend	Cat # 103112
Human Flow Antibodies		
CD45 PE	Biolegend	Cat # 304008
CD19 APCCY7	Biolegend	Cat # 302218
CD33 FITC	Biolegend	Cat # 303304
CD14 PECy5	Biolegend	Cat # 301864
CD16 BV421	Biolegend	Cat # 302038
Lineage cocktail FITC	Biolegend	Cat # 348801
CD34 APCCy7	Biolegend	Cat # 343514
CD38 PECy5	Biolegend	Cat # 303508
CD45RA BV421	Biolegend	Cat # 304130
CD123 PECy7	Biolegend	Cat # 306010
Human Fc receptor blocking solution	Biolegend	Cat # 422302
*I**nhibitors***		
APX3330	Gift from Mark R. Kelley	
Quizartinib (AC220)	Fisher Scientific	67-885-0
(2-hydroxypropyl)-β-cyclodextrin solution	Sigma Aldrich	H5784
Decitabine	Selleckchem	S1200
AML Patient Samples	Dr. Boswell Scott, IUSM, Indianapolis	
Software and Algorithms		
Flow Jo		
GraphPad Prism 7.0		

Supplementary Table2. Data set used for training of hematopoietic cell marker genes

Cell type	Cell type name	GSE14833	GSE27787	GSE656	GSE42519
HSC	Hematopoietic stem cell	4	4	2	4
MPP	Multipotent progenitors	5	2		
NK	Nature Killer cell	2	2	2	
GMP	Granulocyte-myeloid Progenitors	3			5
MEP	Megakaryocyte-erythroid Progenitors	4			2
CLP	Common lymphoid Progenitors	2			
MKP	Megakaryocyte Progenitors	3			
ProB	Pro-B cell	2			
PreB	Pre-B cell	4			
CD4T	CD4+ T cell			2	4
CD8T	CD8+ T cell			2	4
Mono	Monocyte			2	2
Neu	Neutrophil			2	
B	B cell				2
Gra	Granulocyte				2
Erythroid	Erythroid cell				2
CMP	Common Myeloid Progenitor			3	
PM	Promyelocyte				6
MY	Myelocyte				2
MM	Metamyelocyte				3
BC	Band cell bone marrow				4
PMN	Polymorphonuclear Neutrophil				3

Supplementary Table 3A. Overlap of upregulated HSC self-renewal genes

DF vs WT	TF vs WT	TFD vs WT
Hoxa9	Hoxa9	Hoxa9
Hoxb3	Hoxb3	Hoxb3
Hoxa7	Hoxa7	Hoxb4
Cdkn2c	Hoxb4	Mycl
Etv6	Mycl	Etv6
Spi1	Cdkn2c	Vdr
Vdr	Etv6	Zfp467
Zfp467	Spi1	Zfp521
Prdm5	Vdr	Prdm5
Meis1	Glis2	Meis1
Zfp532	Zfp467	Zfp532
	Zfp521	
	Prdm5	

Supplementary Table 3B. Overlap of downregulated HSC self-renewal genes

DF vs WT	TF vs WT	TFD vs WT
Gata1	Gata1	Gata1
Gata3	Gata2	Gata3
Gatad2b	Mycbp	Pbx1
Bmi1	Ezh2	Nr3c2
Ezh2	Myb	
Msi2	Stil	
Nr3c2	Pbx1	
	Msi2	

Supplementary Table 4. Overlap of dysregulated cytokine genes

Upregulated

DF vs WT	TF vs WT	TFD vs WT
Cd34	Anpep	Cd34
Csf1r	Cd34	Csf1r
Csf2ra	Csf1r	Csf2ra
Flt3	Csf2ra	Flt3
Il3ra	Fcgr1	H2-Eb1
Il6ra	Flt3	Il3ra
Itga1	H2-Eb1	Il6ra
Csf2rb	Il3ra	Il6ra
Ifnar2	Il6ra	Itga1
Ifngr1	Itga1	Itgb3
Ifngr2	Itga6	Ifnar2
Il31ra	Ifnar1	Ifngr1
Il6st	Ifnar2	Il10ra
	Ifngr1	Il12rb2
	Ifngr2	Il131ra
	Il10ra	Il6st
	Il12rb2	
	Il131ra	
	Il6st	

Downregulated

DF vs WT	TF vs WT	TFD vs WT
Cd19	Cd19	Cd19
Cd2	Cd2	Cd2
Cd22	Cd24a	Cd22
Cd24a	Cd37	Cd24a
Cd36	Cd38	Cd37
Cd37	Cd55	Cd38
Cd38	Cd8a	Cd3e
Cd3e	Cd8b1	Cd3g
Cd4	Cd9	Cd55
Cd5	Cr2	Cd8a
Cd55	Csf3r	Cd8b1
Cd7	Dntt	Cr2
Cd8b1	Epor	Dntt
Cr2	Fcer2a	Epor
Dntt	Gypa	Fcer2a
Epor	Il1b	Gypa
Fcer2a	Il1r2	Il1b
Gp1ba	Il2ra	Il1r2
Gp5	Il5ra	Il2ra
Gypa	Il6	Il5ra
Il1b	Il7r	Il6
Il1r2	Itga2	Il7r
Il2ra	Itgam	Ms4a1
Il5ra	Ms4a1	Tfrc
Il7r	Tfrc	Cxcr2
Il9r	Cxcr2	Gfra1
Itga2	Gfra1	Gfra2
Ms4a1	Gfra2	Il2rb
Tfrc	Il2rb	
Gfra1		
Gfra2		
Il2rb		
Il2rg		
